# Genetic diversity and chemical variability of *Lippia* spp. (Verbenaceae)

**DOI:** 10.1186/s13104-018-3839-y

**Published:** 2018-10-12

**Authors:** Milene C. Almeida, Ediedia S. Pina, Camila Hernandes, Sonia M. Zingaretti, Silvia H. Taleb-Contini, Fátima R. G. Salimena, Svetoslav N. Slavov, Simone K. Haddad, Suzelei C. França, Ana M. S. Pereira, Bianca W. Bertoni

**Affiliations:** 1 Departamento de Biotecnologia, Universidade de Ribeirão Preto, Ribeirão Preto, São Paulo Brazil; 2 Hospital Israelita Albert Einstein, São Paulo, São Paulo Brazil; 3 Departamento de Botânica, Universidade Federal de Juiz de Fora, Juiz de Fora, Minas Gerais Brazil; 4 Hemocentro de Ribeirão Preto, Faculdade de Medicina de Ribeirão Preto, Universidade de São Paulo, Ribeirão Preto, São Paulo Brazil

**Keywords:** *Lippia origanoides*, Phytomedicine, AFLP, ITS2, CG/MS, Phylogenetic relationships

## Abstract

**Background:**

The genus *Lippia* comprises 150 species, most of which have interesting medicinal properties. *Lippia sidoides* (syn. *L. origanoides*) exhibits strong antimicrobial activity and is included in the phytotherapy program implemented by the Brazilian Ministry of Health. Since species of *Lippia* are morphologically very similar, conventional taxonomic methods are sometimes insufficient for the unambiguous identification of plant material that is required for the production of certified phytomedicines. Therefore, genetic and chemical analysis with chemotype identification will contribute to a better characterization of Lippia species.

**Methods:**

Amplified Length Polymorphism and Internal Transcribed Spacer molecular markers were applied to determine the plants’ genetic variability, and the chemical variability of *Lippia* spp. was determined by essential oil composition.

**Results:**

Amplified Length Polymorphism markers were efficient in demonstrating the intra and inter-specific genetic variability of the genus and in separating the species *L. alba*, *L. lupulina* and *L. origanoides* into distinct groups. Phylogenetic analysis using Amplified Length Polymorphism and markers produced similar results and confirmed that *L. alba* and *L. lupulina* shared a common ancestor that differ from *L. origanoides*. Carvacrol, endo-fenchol and thymol were the most relevant chemical descriptors.

**Conclusion:**

Based on the phylogenetic analysis it is proposed that *L. grata* should be grouped within *L. origanoides* due to its significant genetic similarity. Although Amplified Length Polymorphism and Internal Transcribed Spacer markers enabled the differentiation of individuals, the genotype selection for the production of certified phytomedicines must also consider the chemotype classification that reflects their real medicinal properties.

**Electronic supplementary material:**

The online version of this article (10.1186/s13104-018-3839-y) contains supplementary material, which is available to authorized users.

## Background

The genus *Lippia* comprises 150 species, most of which are distributed in the Neotropical ecozone [1]. Brazil stands out as the centre of diversity of the genus with 98 species presenting high degrees of endemism. More than half of these species are concentrated in the Espinhaço Range, which stretches 1000 km through the Brazilian states of Minas Gerais and Bahia [2]. However, 18 species are considered rare or endangered, and nine are under threat of extinction due to the destruction of their natural environments in the *Cerrado* region (Brazilian type of Savana) [3].

The Brazilian Ministry of Health has developed an extensive phytotherapy program over the last decade with the aim of providing access to herbal medicines for the entire population. One of the target species of this program is *Lippia sidoides* Cham. (syn. *L. origanoides*) (Verbenaceae), a plant that was included in the *Formulário de Fitoterápicos da Farmacopéia Brasileira* [4, 5] based on its strong antimicrobial activity, against *Candida albicans* [6, 7], *Staphylococcus aureus*, and *Escherichia coli* [8] were included due to the presence of terpenoids in the essential oil. It is well known that terpenoids are produced as part of the plant defense system and have been considered a promising source of biological compounds [9–12]. Several essential oil compounds such as linalool, eugenol, carvone, vanillin, carvacrol, and thymol have been accepted by the European Commission to be used in food preservation or flavorings [13].

The morphological similarities between this and other species within the genus tend to complicate the accurate botanical identification, leading to difficulties in the production of certified herbal medicines.

Based on the differential morphological characteristics, the genus *Lippia* was classified in seven sections [14]. The *Zapania* Schauer section is the most complex and exhibits inflorescences with flat bracts, spirally arranged, globose or hemispheric type, capituliform, with varying numbers of chromosomes (2n = 10–28). *L. alba* (Mill.) N.E.Br., *L. aristata* Schauer, *L. brasiliensis* (Link) T.R.S. Silva, *L. corymbosa* Cham., *L. diamantinensis* Glaz., *L. duartei* Moldenke, *L. filifolia* Mart. & Schauer, *L. hermannioides* Cham., *L. lacunosa* Mart. & Schauer, *L. rotundifolia* Cham. and *L. rubella* (Moldenke) T.R.S. Silva & Salimena [15, 16] are among the representatives of this section in the Brazilian flora.

The *Goniostachyum* Schauer section presents tetrastic inflorescences formed by four series of keeled bracts aligned in rows. This section is considered monophyletic and is characterized by small variations (2n = 12) in the number of chromosomes [15, 17]. A recent revision of the species belonging to *Goniostachyum* resulted in the validation of only four representatives, namely: *L. grata* Schauer, *L. origanoides* Kunth, *L. sericea* Cham. and *L. stachyoides* Cham. [17]. Thus, some nominations of species or varieties must be considered synonyms of *L. origanoides* including, amongst others, *L. sidoides*, *L. graveolens* Kunth, *L. microphylla* Cham., *L. salviifolia* Cham., *L. velutina* Schauer, and *Lantana origanoides* Martens & Galeotti. Additionally, *L. dumetorum* Herzog, *L. gracilis* Schauer ex DC, *L. hickenii* Tronc., *L. laxibracteata* Herzog, and others have received the synonym *L. grata*. [17]. The *Rhodolippia* Schauer section comprises species with numbers of chromosomes that are intermediate between those of sections *Zapania* and *Goniostachyum* [15, 18], including *L. bradei* Moldenke, *L. felippei* Moldenke, *L. florida* Cham., *L. hederaefolia* Mart. & Schauer, *L. lupulina* Cham., *L. pseudothea* Schauer, *L. rhodocnemis* Mart. & Schauer, and *L. rosella* Moldenke.

However, the taxonomic classification of *Lippia* remains incoherent mainly due to the morphological variability within the genus and the existence of a great number of nomenclatures for this species resulting in classification dualism, both of which can be explained if we consider the interaction between the genotype and the environment [19]. In this context, studies aimed at evaluating the genetic structure of the genus through analysis of molecular markers could be useful in classifying species into clusters according to their genetic similarities.

A number of reports confirm that the association of molecular markers such as amplified fragment length polymorphism (AFLP) and internal transcribed spacer 2 (ITS2) can contribute significantly to the analysis of genetic variability and phylogenetic inferences [20, 21].

Besides molecular markers, chemical markers can also be used to help the correct plant characterization. WinK [22] developed a phylogenetic classification based on the secondary metabolites produced by Fabaceae, Solanaceae and Lamiacea families. The author considered that the ability or inability to produce a specific metabolite—shown by different members of related phylogenetic groups, are the result of differential expression patterns that reflect specific plant strategies for adaptation that were incorporated into the phylogenetic structure.

Therefore, the aim of the present study was to assess the genetic and chemical variability of species of *Lippia* spp. using molecular and chemical markers, to draw inferences regarding the phylogenetic relationships within the genus, and to identify inconsistencies in the current taxonomic classification for the correct use of those plants in phytomedicine.

## Methods

### Plant materials, DNA extractions, PCR amplifications and sequencing

We used 141 accessions (Table 1) comprising six *Lippia* species; although *L*. *sidoides* and *L. origanoides* are synonymous, they were considered, for the purposes of this study, as they were classified. Thirty-seven of these accessions were from the medicinal plants germplasm bank (Ribeirão Preto University, Brazil) and 104 were collected in the medicinal botanical garden of Nature Pharmacy, Brazil, with voucher numbers; 1340; 1350;1351; 1353; 1355; 1359; 1360; 1362–1365; 1368–1376; 1378–1380; 2000–2015; 2017–2112; 2114; 2471; 2473–2475. Sampling permission, for both locations, were obtained from by the Brazilian Council for the Administration and Management of Genetic Patrimony (CGEN) of the Brazilian Ministry of the Environment (MMA) by the National Council for Scientific and Technological Development (CNPq—CGEN/MMA Process #: 02001.005059/2011-71). Fátima R. G. Salimena (Juiz de Fora Federal University, Brazil) identified all samples. Total genomic DNA was extracted from 0.15 g of frozen leaves using the cetyltrimethylammonium bromide (CTAB) method [23]. The DNA integrity was determined by electrophoresis on 0.8% agarose gels and the concentration and quality of the isolated nucleic acid was determined by a NanoPhotometer^®^ P360 spectrophotometer (Inplen, Westlake Village, CA, USA).

**Table 1:** Location, Geographical coordinates and voucher number of *Lippia* species

Individual	Taxonomic identification	Location (State)	Geographical coordinates	Voucher
LT1	*L. origanoides*	Bahia 1	11°42′31.9″–39°31′10.5″ 222 m	2000
LT2	*L. origanoides*	Bahia 1	11°42′31.9″–39°31′10.5″ 222 m	2001
LT3	*L. origanoides*	Bahia 1	11°42′31.9″–39°31′10.5″ 222 m	2002
LT4	*L. origanoides*	Bahia 1	11°42′31.9″–39°31′10.5″ 222 m	2003
LT5	*L. origanoides*	Bahia 1	11°42′31.9″–39°31′10.5″ 222 m	2007
LT6	*L. origanoides*	Bahia 1	11°42′31.9″–39°31′10.5″ 222 m	2004
LT7	*L. origanoides*	Bahia 1	11°42′31.9″–39°31′10.5″ 222 m	2005
LT8	*L. origanoides*	Bahia 1	11°42′31.9″–39°31′10.5″ 222 m	2006
LT9	*L. grata*	Bahia 1	11°42′31.9″–39°31′10.5″ 222 m	2097
LT10	*L. orig*. × *velut.*	Bahia 1	11°42′31.9″–39°31′10.5″ 222 m	2077
LT11	*L. origanoides*	Bahia 1	11°42′31.9″–39°31′10.5″ 222 m	2008
LT12	*L. origanoides*	Bahia 1	11°42′31.9″–39°31′10.5″ 222 m	2009
LT13	*L. origanoides*	Bahia 1	11°42′31.9″–39°31′10.5″ 222 m	2010
LT14	*L. origanoides*	Bahia 1	11°42′31.9″–39°31′10.5″ 222 m	2011
LT15	*L. origanoides*	Bahia 1	11°42′31.9″–39°31′10.5″ 222 m	2012
LT16	*L. grata*	Bahia 1	11°42′31.9″–39°31′10.5″ 222 m	2098
LT18	*L. origanoides*	Bahia 1	11°42′31.9″–39°31′10.5″ 222 m	2013
LT19	*L. origanoides*	Bahia 1	11°42′31.9″–39°31′10.5″ 222 m	2014
LT20	*L. origanoides*	Bahia 1	11°42′31.9″–39°31′10.5″ 222 m	2015
LT23	*L. orig*. × *velut.*	Bahia 1	11°42′31.9″–39°31′10.5″ 222 m	2078
LT24	*L. origanoides*	Bahia 1	11°42′31.9″–39°31′10.5″ 222 m	2079
LT26	*L. origanoides*	Bahia 1	11°42′31.9″–39°31′10.5″ 222 m	2017
LT27	*L. origanoides*	Bahia 1	11°42′31.9″–39°31′10.5″ 222 m	2018
LT30	*L. origanoides*	Bahia 1	11°42′31.9″–39°31′10.5″ 222 m	2019
LT31	*L. origanoides*	Bahia 1	11°42′31.9″–39°31′10.5″ 222 m	2020
LT32	*L. origanoides*	Bahia 1	11°42′31.9″–39°31′10.5″ 222 m	2021
LT33	*L. origanoides*	Bahia 1	11°42′31.9″–39°31′10.5″ 222 m	2022
LT34	*L. origanoides*	Bahia 1	11°42′31.9″–39°31′10.5″ 222 m	2023
LT35	*L. origanoides*	Bahia 1	11°42′31.9″–39°31′10.5″ 222 m	2024
LT36	*L. origanoides*	Bahia 1	11°42′31.9″–39°31′10.5″ 222 m	2025
LT38	*L. origanoides*	Bahia 1	11°42′31.9″–39°31′10.5″ 222 m	2026
LT40	*L. orig*. × *velut.*	Bahia 1	11°42′31.9″–39°31′10.5″ 222 m	2080
LT42	*L. velutina*	Bahia 1	11°42′31.9″–39°31′10.5″ 222 m	2096
LT43	*L. origanoides*	Bahia 1	11°42′31.9″–39°31′10.5″ 222 m	2027
LT44	*L. grata*	Bahia 1	11°42′31.9″–39°31′10.5″ 222 m	2099
LT45	*L. origanoides*	Bahia 1	11°42′31.9″–39°31′10.5″ 222 m	2028
LT46	*L. velutina*	Bahia 1	11°42′31.9″–39°31′10.5″ 222 m	2095
LT47	*L. grata*	Bahia 1	11°42′31.9″–39°31′10.5″ 222 m	2100
LT48	*L. origanoides*	Bahia 1	11°42′31.9″–39°31′10.5″ 222 m	2029
LT49	*L. origanoides*	Bahia 1	11°42′31.9″–39°31′10.5″ 222 m	2030
LT50	*L. origanoides*	Minas Gerais 3	19°82′02.2″–43°91′96.9″ 589 m	2031
LT51	*L. orig*. × *velut.*	Minas Gerais 3	19°82′02.2″–43°91′96.9″ 589 m	2081
LT52	*L. origanoides*	Bahia 2	10°33′38.1″–40°16′37.7″ 489 m	2032
LT53	*L. origanoides*	Bahia 2	10°33′38.1″–40°16′37.7″ 489 m	2033
LT54	*L. orig*. × *velut.*	Bahia 2	10°33′38.1″–40°16′37.7″ 489 m	2082
LT55	*L. origanoides*	Bahia 2	10°33′38.1″–40°16′37.7″ 489 m	2034
LT57	*L. origanoides*	Bahia 2	10°33′38.1″–40°16′37.7″ 489 m	2035
LT59	*L. origanoides*	Bahia 2	10°33′38.1″–40°16′37.7″ 489 m	2036
LT60	*L. origanoides*	Bahia 2	10°33′38.1″–40°16′37.7″ 489 m	2037
LT61	*L. origanoides*	Bahia 2	10°33′38.1″–40°16′37.7″ 489 m	2038
LT63	*L. orig*. × *velut.*	Bahia 2	10°33′38.1″–40°16′37.7″ 489 m	2083
LT64	*L. origanoides*	Bahia 2	10°33′38.1″–40°16′37.7″ 489 m	2039
LT65	*L. origanoides*	Bahia 2	10°33′38.1″–40°16′37.7″ 489 m	2040
LT66	*L. origanoides*	Bahia 2	10°33′38.1″–40°16′37.7″ 489 m	2041
LT67	*L. origanoides*	Bahia 2	10°33′38.1″–40°16′37.7″ 489 m	2042
LT68	*L. orig*. × *velut.*	Bahia 2	10°33′38.1″–40°16′37.7″ 489 m	2084
LT69	*L. orig*. × *velut.*	Bahia 2	10°33′38.1″–40°16′37.7″ 489 m	2085
LT70	*L. origanoides*	Bahia 2	10°33′38.1″–40°16′37.7″ 489 m	2043
LT71	*L. origanoides*	Bahia 2	10°33′38.1″–40°16′37.7″ 489 m	2044
LT72	*L. origanoides*	Bahia 2	10°33′38.1″–40°16′37.7″ 489 m	2045
LT73	*L. origanoides*	Bahia 2	10°33′38.1″–40°16′37.7″ 489 m	2046
LT75	*L. origanoides*	Bahia 2	10°33′38.1″–40°16′37.7″ 489 m	2047
LT76	*L. origanoides*	Bahia 2	10°33′38.1″–40°16′37.7″ 489 m	2048
LT77	*L. orig*. × *velut.*	Bahia 2	10°33′38.1″–40°16′37.7″ 489 m	2086
LT78	*L. velutina*	Bahia 2	10°33′38.1″–40°16′37.7″ 489 m	2094
LT79	*L. orig*. × *velut.*	Bahia 2	10°33′38.1″–40°16′37.7″ 489 m	2087
LT80	*L. origanoides*	Bahia 2	10°33′38.1″–40°16′37.7″ 489 m	2049
LT81	*L. origanoides*	Bahia 2	10°33′38.1″–40°16′37.7″ 489 m	2050
LT82	*L. origanoides*	Bahia 2	10°33′38.1″–40°16′37.7″ 489 m	2051
LT83	*L. origanoides*	Bahia 2	10°33′38.1″–40°16′37.7″ 489 m	2052
LT86	*L. orig*. × *velut.*	Bahia 2	10°33′38.1″–40°16′37.7″ 489 m	2088
LT87	*L. origanoides*	Bahia 2	10°33′38.1″–40°16′37.7″ 489 m	2053
LT88	*L. orig*. × *velut.*	Bahia 2	10°33′38.1″–40°16′37.7″ 489 m	2089
LT89	*L. velutina*	Bahia 2	10°33′38.1″–40°16′37.7″ 489 m	2093
LT90	*L. origanoides*	Bahia 2	10°33′38.1″–40°16′37.7″ 489 m	2054
LT91	*L. orig*. × *velut.*	Bahia 2	10°33′38.1″–40°16′37.7″ 489 m	2090
LT92	*L. origanoides*	Bahia 2	10°33′38.1″–40°16′37.7″ 489 m	2055
LT93	*L. origanoides*	Bahia 2	10°33′38.1″–40°16′37.7″ 489 m	2056
LT94	*L. origanoides*	Bahia 2	10°33′38.1″–40°16′37.7″ 489 m	2057
LT96	*L. origanoides*	Bahia 2	10°33′38.1″–40°16′37.7″ 489 m	2058
LT97	*L. origanoides*	Bahia 2	10°33′38.1″–40°16′37.7″ 489 m	2059
LT98	*L. origanoides*	Bahia 2	10°33′38.1″–40°16′37.7″ 489 m	2060
LT99	*L. origanoides*	Bahia 2	10°33′38.1″–40°16′37.7″ 489 m	2061
LT100	*L. origanoides*	Bahia 2	10°33′38.1″–40°16′37.7″ 489 m	2062
LT101	*L. origanoides*	Bahia 2	10°33′38.1″–40°16′37.7″ 489 m	2063
LT102	*L. origanoides*	Bahia 2	10°33′38.1″–40°16′37.7″ 489 m	2064
LT103	*L. origanoides*	Bahia 2	10°33′38.1″–40°16′37.7″ 489 m	2065
LT104	*L. origanoides*	Bahia 2	10°33′38.1″–40°16′37.7″ 489 m	2066
LT105	*L. origanoides*	Bahia 2	10°33′38.1″–40°16′37.7″ 489 m	2067
LT107	*L. origanoides*	Bahia 2	10°33′38.1″–40°16′37.7″ 489 m	2068
LT108	*L. origanoides*	Bahia 2	10°33′38.1″–40°16′37.7″ 489 m	2069
LT109	*L. origanoides*	Bahia 2	10°33′38.1″–40°16′37.7″ 489 m	2070
LT110	*L. origanoides*	Bahia 2	10°33′38.1″–40°16′37.7″ 489 m	2071
LT111	*L. origanoides*	Bahia 3	11°11′ 25.5″–39°25′39.5″ 344 m	2072
LT112	*L. origanoides*	Bahia 3	11°11′ 25.5″–39°25′39.5″ 344 m	2073
LT113	*L. origanoides*	Bahia 3	11°11′ 25.5″–39°25′39.5″ 344 m	2075
LT114	*L. origanoides*	Bahia 3	11°11′ 25.5″–39°25′39.5″ 344 m	2074
LT115	*L. origanoides*	Bahia 3	11°11′ 25.5″–39°25′39.5″ 344 m	2076
LT116	*L. orig*. × *velut.*	São Paulo	21°11′55.5″–47°44′08.8″ 566 m	2091
LT117	*L. orig*. × *velut.*	São Paulo	21°11′55.5″–47°44′08.8″ 566 m	2092
LT118	*L. origanoides*	Minas Gerais 1	19°36′49.9″–42°08′20.8″ 929 m	2110
LT120	*L. alba*	São Paulo	21°11′55.5″–47°44′08.8″ 566 m	2101
LT121	*L. alba*	São Paulo	21°11′55.5″–47°44′08.8″ 566 m	2102
LT122	*L. alba*	São Paulo	21°11′55.5″–47°44′08.8″ 566 m	2103
LT123	*L. alba*	São Paulo	21°11′55.5″–47°44′08.8″ 566 m	2104
LT124	*L. alba*	São Paulo	21°11′55.5″–47°44′08.8″ 566 m	2105
LT125	*L. alba*	São Paulo	21°11′55.5″–47°44′08.8″ 566 m	2106
LT126	*L. alba*	Minas Gerais 2	19°51′37.3″–47° 20 27.9″1069 m	2106
LT127	*L. alba*	Minas Gerais 1	19°36′49.9″–42°08′20.8″ 929 m	2108
LT128	*L. alba*	Minas Gerais 1	19°36′49.9″–42°08′20.8″ 929 m	2109
LU129	*L. orig*. × *velut.*	Bahia 4	10°31′14.8″–40°13′57.7″ 594 m	1364
LU130	*L. orig*. × *velut.*	Bahia 5	10°50′48.1″–39°35′45.0″ 358 m	1380
LU132	*L. orig*. × *velut.*	Bahia 3	11°11′25.5″–39°25′39.5″ 344 m	1350
LU133	*L. orig*. × *velut.*	Bahia 3	11°11′25.5″–39°25′39.5″ 344 m	1351
LU134	*L. origanoides*	Bahia 3	11°11′25.5″–39°25′39.5″ 344 m	1353
LU135	*L. origanoides*	Bahia 3	11°11′25.5″–39°25′39.5″ 344 m	1355
LU137	*L. origanoides*	Bahia 3	11°11′25.5″–39°25′39.5″ 344 m	1359
LU138	*L. origanoides*	Bahia 3	11°11′25.5″–39°25′39.5″ 344 m	1360
LU140	*L. origanoides*	Bahia 3	11°11′25.5″–39°25′39.5″ 344 m	1362
LU141	*L. origanoides*	Bahia 3	11°11′25.5″–39°25′39.5″ 344 m	1363
LU142	*L. grata*	Bahia 6	11°11′25.5″–39°25′39.5″ 344 m	2475
LU143	*L. grata*	Bahia 6	11°11′25.5″–39°25′39.5″ 344 m	2474
LU144	*L. grata*	Bahia 6	11°11′25.5″–39°25′39.5″ 344 m	2473
LU145	*L. velutina*	Ceará 1	03°69′79.3″–38°57′35.1″ 005 m	2111
LU146	*L. velutina*	Ceará 1	03°69′79.3″–38°57′35.1″ 005 m	2112
LU148	*L. velutina*	Ceará 1	03°69′79.3″–38°57′35.1″ 005 m	2114
LU150	*L. orig*. × *velut.*	Bahia 1	11°42′31.9″–39°31′10.5″ 222 m	1365
LU151	*L. orig*. × *velut.*	Bahia 1	11°42′31.9″–39°31′10.5″ 222 m	1366
LU153	*L. orig*. × *velut.*	Bahia 1	11°42′31.9″–39°31′10.5″ 222 m	1368
LU154	*L. orig*. × *velut.*	Bahia 1	11°42′31.9″–39°31′10.5″ 222 m	1369
LU155	*L. orig*. × *velut.*	Bahia 1	11°42′31.9″–39°31′10.5″ 222 m	1370
LU156	*L. orig*. × *velut.*	Bahia 1	11°42′31.9″–39°31′10.5″ 222 m	1371
LU157	*L. orig*. × *velut.*	Bahia 1	11°42′31.9″–39°31′10.5″ 222 m	1372
LU158	*L. orig*. × *velut.*	Bahia 1	11°42′31.9″–39°31′10.5″ 222 m	1373
LU159	*L. orig*. × *velut.*	Bahia 1	11°42′31.9″–39°31′10.5″ 222 m	1374
LU160	*L. orig*. × *velut.*	Bahia 1	11°42′31.9″–39°31′10.5″ 222 m	1375
LU161	*L. origanoides*	Bahia 1	11°42′31.9″–39°31′10.5″ 222 m	1376
LU162	*L. orig*. × *velut.*	Bahia 1	11°42′31.9″–39°31′10.5″ 222 m	1378
LU163	*L. orig*. × *velut.*	Bahia 1	11°42′31.9″–39°31′10.5″ 222 m	1379
LU164	*L. grata*	Ceará 2	03°80′41.1″–08°45′60.7″ 014 m	2471
LU165	*L. lupulina*	Minas Gerais 2	19°51′37.3″–47°20′27.9″1069 m	1340

Location: Bahia 1: Riachão do Jacuípe; Bahia 2: Campo Formoso; Bahia 3: Santa Luz; Bahia 4: Missão; do Sahy; Bahia 5: Queimadas; Bahia 6: Contagem; Ceará 1: Quatro Varas; Ceará 2: Orto Fortaleza; Minas Gerais 1: Araxá; Minas Gerais 2: Sacramento; Minas Gerais 3: Mateus Leme; São Paulo: Jardinópolis

### Reactions and analysis of AFLP data

Samples from all 141 genotypes were analyzed according to the method of Vos et al. [24]. Briefly, genomic DNA (300 ng) was digested with *Eco*RI/*Mse*I enzymes (New England Biolabs, Ipswich, MA, US) at 37 °C for 3 h, followed by inactivation at 70 °C for 5 min. Resulting DNA fragments were ligated to adaptors complementary to the restriction enzymes recognition sites and the ligation products were then diluted 6× with deionized water. In the first round of polymerase chain reaction (PCR), pre-selective amplification was achieved with primers *Eco*RI + 1 (50 µM) and *Mse*I + 1 (50 µM). The pre-selective products were diluted 10× with deionized water and a second round of PCR was carried out using marker primers fluorescently tagged with IRDye^®^ (LI-COR Biosciences, Lincoln, NE, USA). The selected marked primers were those that generated the largest number of polymorphic bands. Genotyping of individuals was performed using a 4300 DNA Analyzer (LI-COR Biosciences, Lincoln, NE, USA) while data alignment was accomplished with the aid of Saga^MX^ Automated AFLP Analysis software version 3.3 guided by molecular weight markers in the range 50–700 bp. A binary matrix was constructed based on a 1/0 score for the presence/absence of each electrophoretic band. The genetic distance was calculated from the binary matrix using Jaccard indices, whereas the dendrogram was constructed using the unweighted pair group method with arithmetic average (UPGMA) clustering technique with 1000 permutations and Free Tree software version 0.9.1.50 [25] and visualized through TreeView X program [26]. The genetic structure of genotypes was established by principal coordinates analysis (PCoA) using the software GenAlEx version 6.5 [27] and STRUCTURE version 2.2.4 [28], which generated a posterior distribution based on Bayesian and admixture models. Each analysis comprised a “burn-in” of 200,000 interactions followed by a run length of 500,000 interactions and five independent runs for each *K* value (*K* = 1 to 7). The most probable number of genetic groups was determined from the Δ*K* value [29]. The correlation between genetic and geographical data was performed using the Mantel test and the POPGENE 32 [30] and GENES version 2009.7.0 [31] programs with 1000 simulations.

### Sequencing and phylogenetic analysis of the ITS2 gene

The primers employed in the amplification reactions ITS2F-5′AATTGCAGAATCCCGTGAAC3′ and ITS2R-5′GGTAATCCCGCCTGACCT3′ were designed based on ITS2 sequences of some Verbenaceae species from the GenBank database at the National Center for Biotechnology Information (NCBI), namely *Aloysia gratissima* (DQ463782.1), *A. gratissima var. schulziae* (AY178651.1), *A. triphylla* (EU761080.1), *Lippia alba* (EU761076.1), *L. alba* (EU761078.1), *L. salsa* (FJ867399.1), and *Phyla dulcis* (EU761079.1). Polymerase chain reaction was performed as described by Chen et al. [32] and the resulting amplified fragments were sequenced using a Thermo Sequenase™ Cycle Sequencing kit (Affymetrix, Inc, Cleveland, USA), following manufacturer recommendations, with e-Seq™ DNA Sequencing version 3.1 (LI-COR Biosciences). Consensus sequences were assembled with the aid of LI-COR Biosciences AlignIR software (version 2.1) and aligned with ClustalW. The sequence alignments were edited using the BioEdit software (version 7.2) [33]. Phylogenetic trees were inferred by the NJ method based on the Kimura-2 parameter using PHYLIP software version 3.69 [34]. The alignment quality of the final phylogenetic tree was verified by the presence of saturation of the nucleotide substitutions, and sequences exhibiting high genetic similarity were excluded from the phylogenetic analysis using DAMBE software version 4.0.36 [35]. Thirty-three sequences of the ITS2 region deposited in the NCBI GenBank were selected as references (Table 2).

**Table 2:** Accession number for ITS2 references of region from NCBI and used code

Species	Code^a^	Accession number
*Lantana micrantha*	Lamicr	HM120854.1
*Lantana angustifolia*	Laangu	HM120857.1
*Lantana scabrida*	Lascab	HM120860.1
*Lantana camara*	Lacama	AF437858.1
*Lantana* sp.	LaspX1	EF190037.1
*Lantana strigocamara*	Lastri	FJ004800.1
*Lantana hodgei*	Lahodg	HM120851.1
*Lantana strigocamara*	LastrA	HM120861.1
*Glandularia subincana*	Glsubi	FJ867442.1
*Glandularia gooddingii var. gooddingii*	Glgvgo	FJ867437.1
*Glandularia guaranitica*	Glguar	FJ867434.1
*Glandularia mendocina*	Glmend	FJ867421.1
*Glandularia dissecta*	Gldiss	FJ867419.1
*Glandularia aristigera*	Glaris	FJ867424.1
*Glandularia cheitmaniana*	Glchei	FJ867444.1
*Glandularia bipinnatifida*	Glbipi	JN686504.1
*Glandularia chiricahensis*	Glchir	FJ867436.1
*Glandularia gooddingii var. nepetifolia*	Glgvne	FJ867439.1
*Glandularia wrightii*	Glwrig	AY928525.1
*Glandularia aurantiaca*	Glaura	FJ867427.1
*Glandularia bipinnatifida*	GlbipT	Fj867440.1
*Glandularia araucana*	Glarau	FJ867429.1
*Glandularia microphulla*	Glmicr	FJ867432.1
*Junellia micrantha*	Jumicr	FJ867462.1
*Junellia caespitosa*	Jucaes	FJ867466.1
*Junellia selaginoides*	Jusela	FJ867463.1
*Junellia aspera var. longidentata*	Juavlo	FJ867460.1
*Junellia spathulata*	Juspat	FJ867456.1
*Junellia ligustima var. lorentzii*	Julvlo	FJ867568.1
*Junellia uniflora*	Juunif	FJ867450.1
*Junellia asparagoides*	Juaspa	FJ867458.1
*Junellia aspera*	Juaspe	FJ867459.1
*Phyla canensis*		HM193969.1

^a^Code used in the phylogenetic tree

### Extraction and analysis of essential oils

The essential oils of *L. origanoides*, *L. origanoides* × *velutina*, *L. velutina*, *L. sidoides*, *L. salviifolia*, and *L. grata* were extracted from dried leaves and flowers by steam distillation in a Clevenger apparatus. A mixture of essential oil/ethyl acetate (v/4v) was analysed using gas chromatograph Varian, model 3900 (Palo Alto, CA, USA), coupled with a Saturn 2100T ion trap mass selective detector and equipped with a non-polar DB-5 fused silica capillary column (30 m × 0.25 mm i.d.; 0.25 μm). The analytical conditions were: carrier gas helium at 1 mL/min; oven temperature 60 to 240 °C at 3 °C/min; injector temperature 240 °C; detector temperature 230 °C; injector split ratio 1:20; injection volume 1 μL; ionization voltage 70 eV. Individual components of oil samples were identified from their Kovats retention indices [36] and by comparison of their electron impact spectra with entries in the NIST62 mass spectral library embedded in the GC/MS system. Data were submitted for principal component analysis (PCA) using the program GENES version 2009.7.0 [31] in order to determine which of the chemical descriptors contributed most to the variability.

## Results

### Analysis based on AFLP markers

**Fig. 1:**
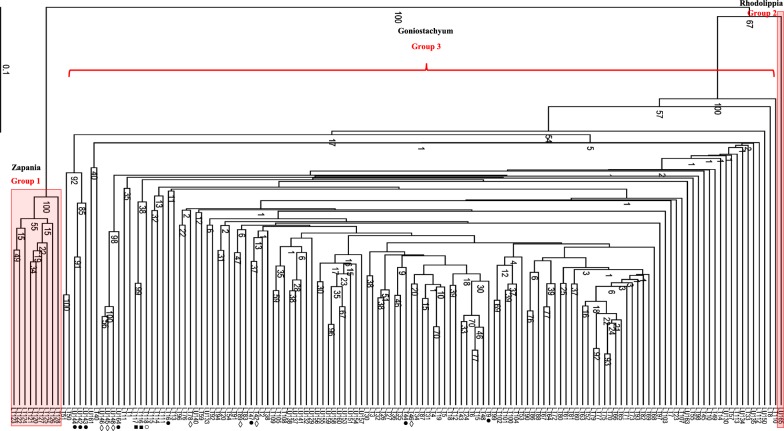
UPGMA dendrogram constructed using data obtained AFLP polymorphic markers (1000 permutations). Individuals featured: Black circle: *L. grata* (LT9, LT16, LT44, LT47, LU142, LU143, LU144); white circle: *L. salvifolia* (LT118); black small circle: *L. sidoides* (LT116; LT117); lozenge: *L. velutina* (LT42, LT46, LT78, LT89, LU145, LU146, LU148)

The set of six primers selected for AFLP analysis of the 141 genotypes amplified 273 loci, of which 267 (97.8%) were polymorphic (Table 3). The dendrogram constructed from these amplified loci (Fig. 1) enabled the 141 genotypes to be discriminated into three distinct genotypic groups, namely group 1 (*L. alba*), group 2 (*L. lupulina*) and group 3 (*L. origanoides*, *L. origanoides* × *velutina*, *L. velutina*, *L. sidoides*, *L. salviifolia*, and *L. grata*). Interestingly, *L. alba* appeared to be more closely related to *L. lupulina* (boostrap 100%) than to *L. origanoides*.

**Table 3:** Sequences of selected primers IRDye 700/800 and number of amplified fragments

Primer	Fragments total	Polymorphic fragments	(%) polymorphic fragments
IRDye 700			
E-AAT-M-AGG	45	44	97.8
E-AAT-M-TC	45	45	100
E-ATG-M-TCG	50	50	100
IRDye 800			
E-AGA-M-AT	17	16	94.1
E-AGA-M-TA	70	68	97.1
E-AG-M-TTC	46	44	95.6
Total	273	267	97.8%

The cluster formed by group 3 indicated the absence of significant differentiation between *L. origanoides*, *L. origanoides* × *velutina*, *L. velutina*, *L. sidoides*, *L. salviifolia*, and *L. grata.* However, only 29% of the hybrid individuals clustered together, whereas 71% assembled with other species. Furthermore, only 37.5% of *L. grata* individuals clustered together, while 62.5% clustered with other species, demonstrating the occurrence of intra- and inter-specific similarities in *Lippia*.

**Fig. 2:**
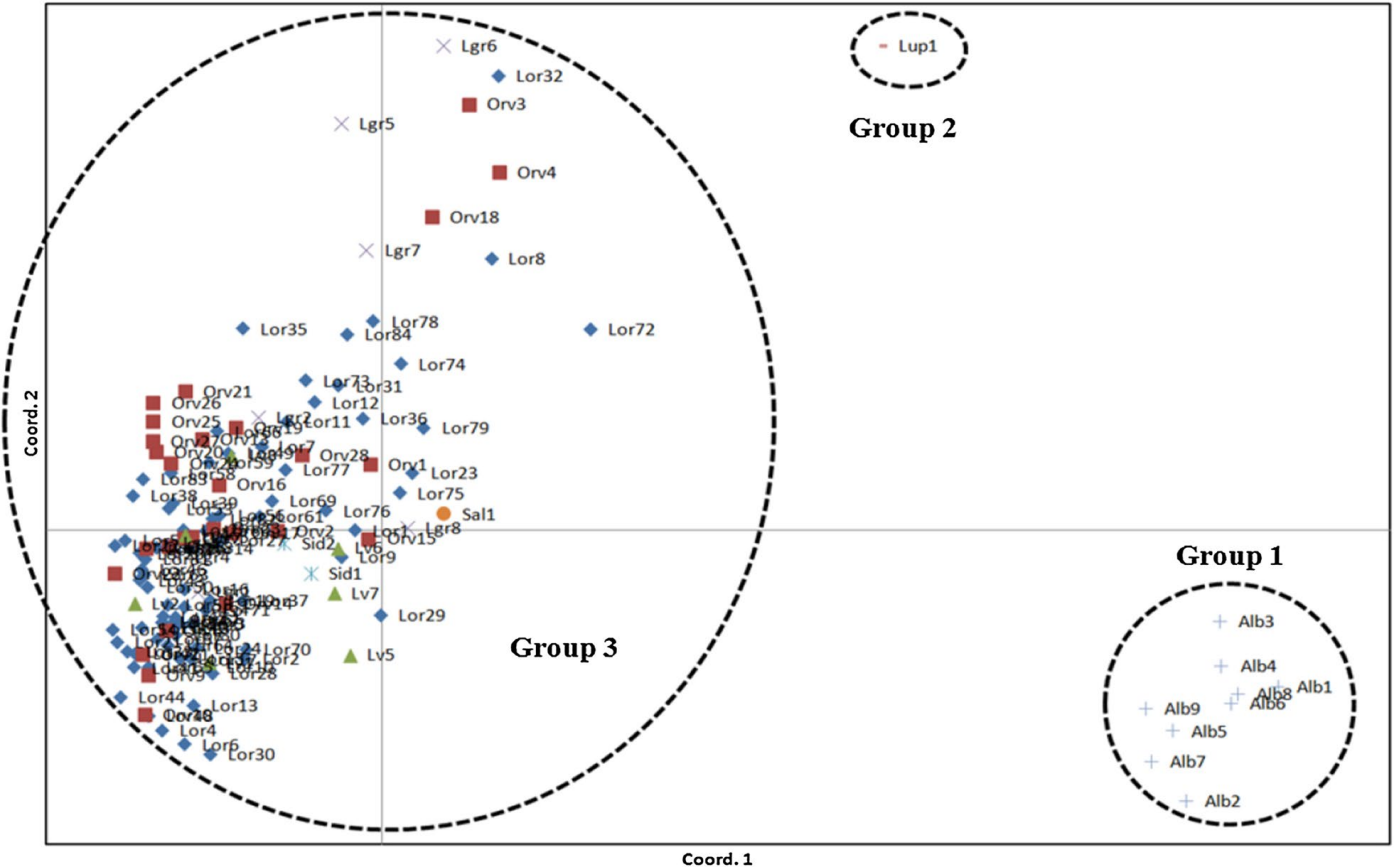
Population structure as determined by principal coordinates analysis (PCoA) of 141 individuals of *Lippia* spp. Group 1—(Alb) *L. alba*; Group 2—(Lup) *L. lupulina*; Group 3—(Lor) *L. origanoides*, (Orv) *L. origanoides* × *velutina*, (Lv) *L. velutina*, (Sid) *L. sidoides*, (Sal) *L. salviifolia* and (Lgr) *L. grata*

**Fig. 3:**
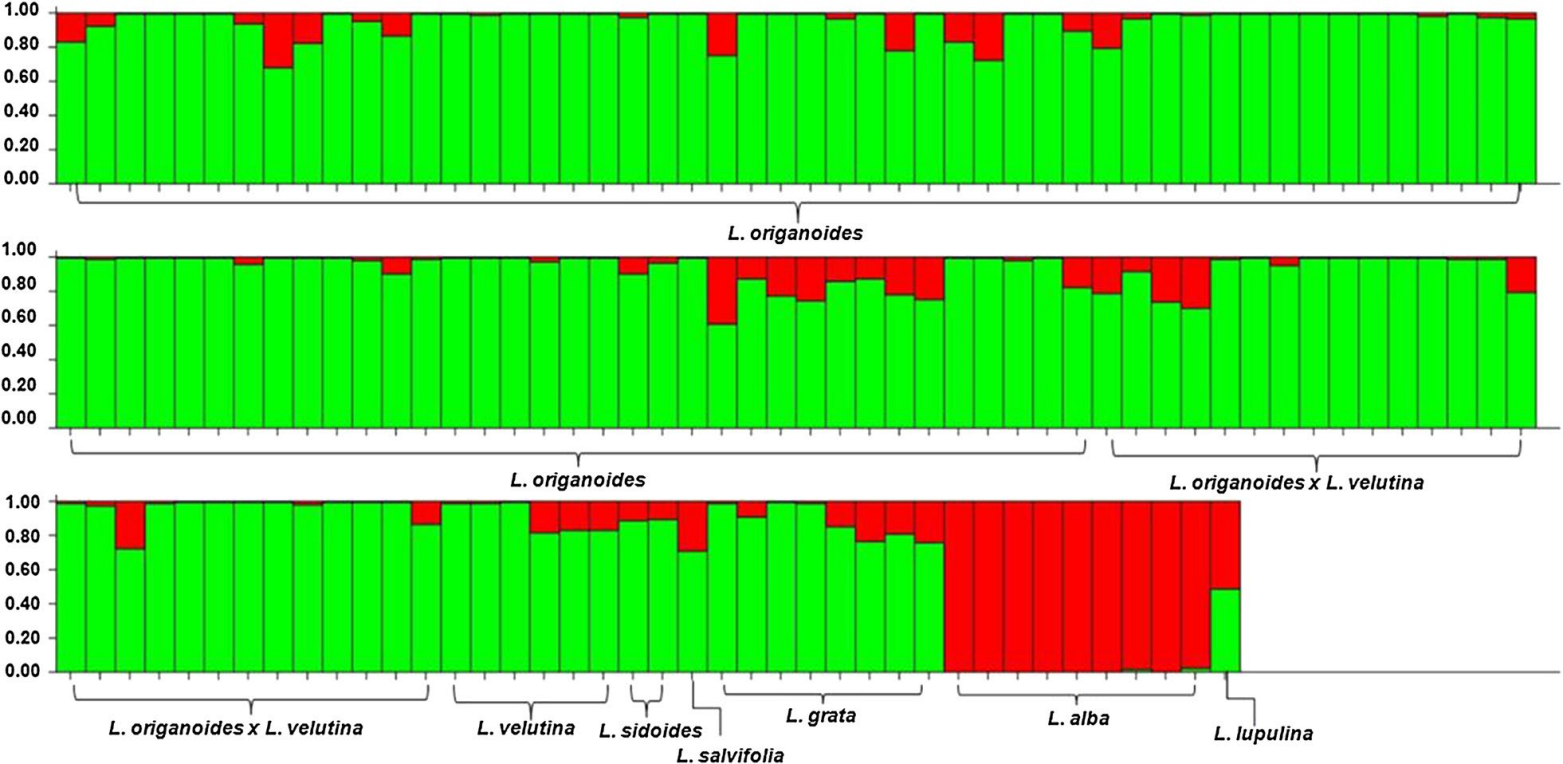
Population structure as determined by Bayesian analysis of 141 individuals genotypes of *Lippia* spp. Individual genotypes are represented by columns while the clusters (*K *= 2) are represented by the colors green and red. Two colors shown for the same individual indicate the percentages of the genome shared between the two groups

The results generated by PCoA analysis also revealed three groups (Fig. 2), but the Bayesian approach using the STRUCTURE software indicated that the genotypes could be organized into two main groups (*K* = 2), suggesting that *L. lupulina* (group 1) occupied an intermediate position between groups 1 and 3 (Fig. 3).

The measure of shared variance between the genetic and geographic variables for individuals in group 3 showed a significant positive correlation (r = 0.80; *p* = 0.46), while the isolation by distance showed the existence of gene flow across group 3 (*Nm* = 1.6), although gene flow between groups 1 and 3 was lower (*Nm *= 1.3).

### Analysis based on ITS2 genotyping

Primers ITS2F and ITS2R amplified DNA fragments of approximately 340 bp. The saturation test revealed that the ITS2 region presents significant genetic variability among the *Lippia* spp.

**Fig. 4:**
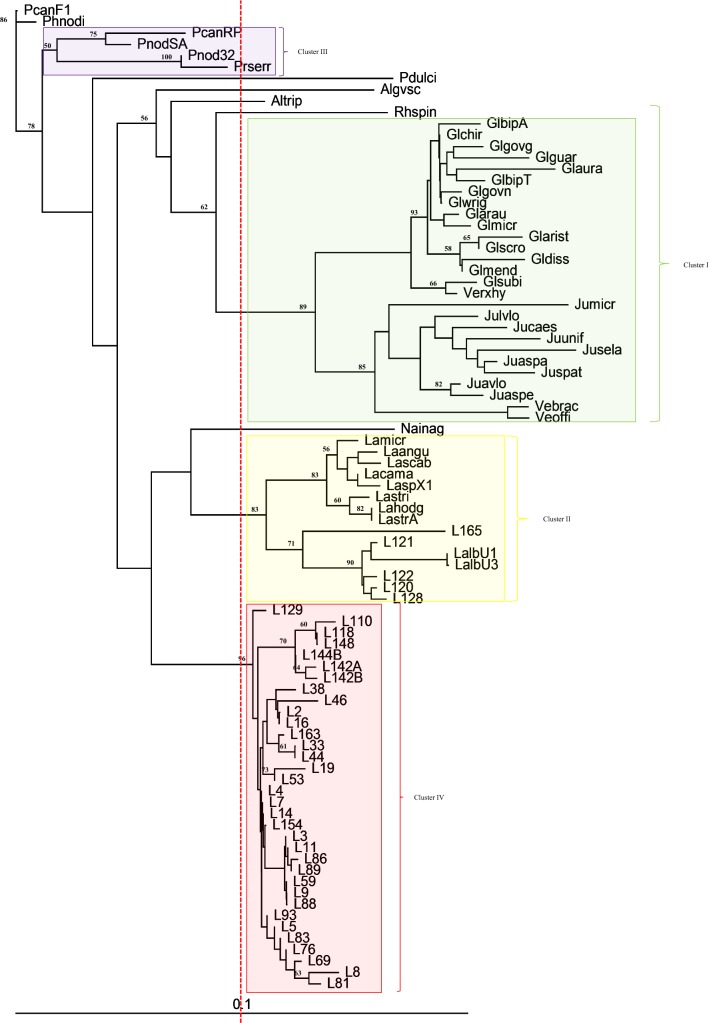
Evolutionary relationships between *Lippia* individuals generated from NJ analysis of ITS2 sequences (Kimura-2 model: PHYLIP software version 3.69). Reference sequences (see Table 2): Lamicr, Laangu, Lascab, Lacama, LaspX1, Lastri, Lahodg, LastrA, Glsubi, Glgvgo, Glguar, Glmend, Gldiss, Glaris, Glchei, Glbipi, Glchir, Glgvne, Glwrig, Glaura, GlbipT, Glarau, Glmicr, Jumicr, Jucaes, Jusela, Juavlo, Juspat, Julvlo, Juunif, Juaspa, Juaspe, *Phylla canensis*. Samples grouped by high genetic similarity: L2, L3, L4, L9, L11, L69, L118, L120, L129, L142 (see Table 4). Capital letters adjacent to code numbers 142 and 144 refer to the amplified bands of 340 bp (A) and 360 bp (B)

The Neighbor-Joining (NJ) of the phylogenetic tree was rooted using the *Phyla canescens* species identified in France (Fig. 4: Table 4). The use of a outgroup species from a different geographic location favors a more robust separation of the tree branches confirming the separation of the phylogenetic groups.

**Table 4:** *Lippia* individual grouped by genetic similarity (ITS2) by DAMBE program version 4.0.36

Individuals with high genetic similarity	Code^a^
LT2, LT31, LT34, LT36: *L. origanoides* LT47: *L. grata* LU156: *L. orig*. × *velut.*	L2
LT3, LT6, LT45: *L. origanoides*	L3
LT4, LT26, LT52, LT73: *L. origanoides* LT116: *L. orig*. × *velut.*	L4
LT7, LT20, LT27, LT32, LT55, LT57, LT60, LT61, LT65, LT66, LT70, LT71, LT75, LT80, LT82, LT87, LT94, LT97, LT98, LT100, LT105, LT107, LT108, LT109, LU137: *L. origanoides* LT10, LT68, LT77, LT79, LT63, LT117, LU130, LU151, LU153, LU158: *L. orig*. × *velut.* LT42, LT78: *L. velutina*	L7
LT9: *L. grata* LT23: *L. orig*. × *velut.* LT90, LT92: *L. origanoides*	L9
LT1, LT11, LT12, LT15, LT24, LT30, LT35, LT43, LT48, LT49, LT64, LT67, LT72, LT104, LU141: *L. origanoides* LT54, LU133: *L. orig*. × *velut.*	L11
LT14, LU155—*L. origanoides*	L14
LT69, LU132: *L. orig*. × *velut.*	L69
LT118, LU145, LU146: *L. velutina* LU164: *L. grata*	L118
LT120, LT123, LT124, LT125, LT126, LT127: *L. alba*	L120
LU129, LU159: *L. orig*. × *velut.*	L129
LU142, LU143: *L. grata*	L142
LU154, LU157: *L. origanoides*	L154

^a^Code used in the phylogenetic tree

The phylogenetic analysis based on the species from the genus *Lantana* (A), *Glandularia* (B), *Junellia* (C), and *Lippia* (D) demonstrated separation of the three branches into four principal clusters with 83%, 93%, 85%, and 96% bootstrap, respectively. In the *Lantana* group, *Lippia lupulina* (L165) and *Lippia alba* (L120, L121, L122, L128), divided into subgroups with a bootstrap of 71% and 83%, respectively, were also identified. The group of *Glandularia* and *Junellia* was clearly subdivided into two groups: one belonging to the species of *Glandularia* and another to the *Junellia* subgroup.

Most of the analyzed species were separated within the *Lippia* group as a monophyletic group. Samples LU145 (*L. velutina*) and LT118 (*L. salviifolia*) were identical to the sample classified as *L. grata* (LU164). Furthermore, a sample classified as *L. velutina* (LT78) was identical to one of *L. sidoides* (LT117), as well as to samples of *L. origanoides* and *L. origanoides* × *velutina*. Additionally, a *L. grata* individual (LT47) was identical to one *L. origanoides* × *velutina* (LU156) and to some *L. origanoides* (LT2, LT31, LT34, LT36).

### Principal Components Analysis (PCA) of essential oil profiles

**Fig. 5:**
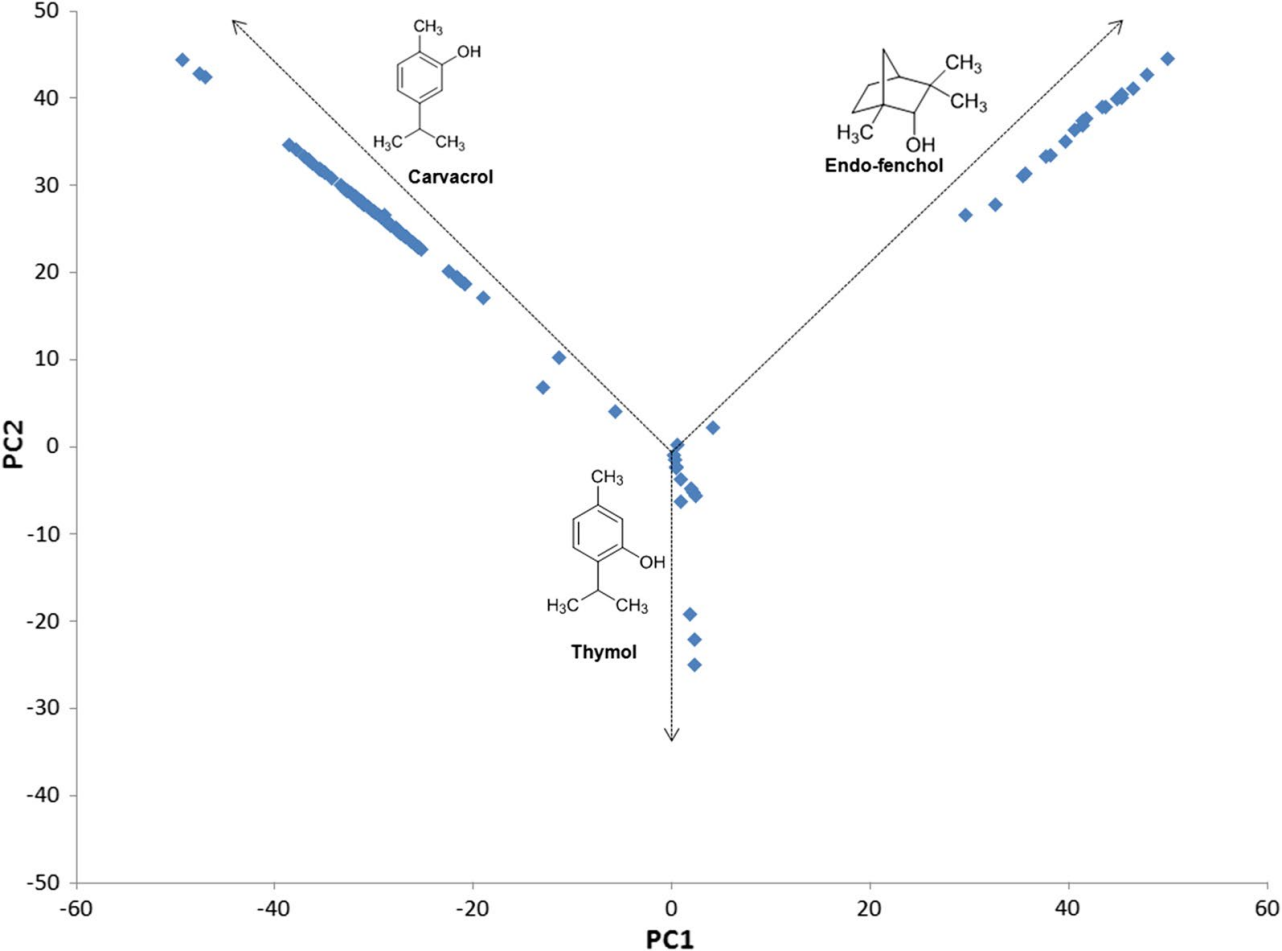
Principal component analysis of the chemical constituents of *Lippia* essential oil

The application of PCA analysis allowed individuals to be grouped according to their different chemical profiles and enabled the seven original chemical descriptors, namely carvacrol, *endo*-fenchol, thymol, β-caryophyllene, isoborneol, *trans*-caryophyllene, and bicyclogermacrene, to be reduced to the first three (Fig. 5). *Endo*-fenchol (PC1) and carvacrol (PC2) accounted for most of the total variation (86.36%), with the first and second components contributing factors of 0.69 and 0.17, respectively, while the contribution of thymol was minimal (only 0.063). Considering all the analyzed individuals, 72% contained carvacrol and 16% contained *endo*-fenchol; since no individuals contained both carvacrol and *endo*-fenchol, the quantification of these two components would cover 88% of the analyzed samples (Fig. 5).

## Discussion

### AFLP analysis

The employed AFLP technique distributed the 141 *Lippia* genotypes into three groups (Fig. 1) that were compatible with the existing taxonomic sections, namely *Zapania* (*L. alba*), *Rhodolippia* (*L. lupulina*) and *Goniostachyum* (*L. origanoides*, *L. sidoides*, *L. salviifolia*, *L. origanoides* × *velutina*, and *L. grata*) [16, 17]. The efficiency of dominant AFLP markers to regroup genetically similar species has been also demonstrated in a number of studies [37–39], having been attributed to the large numbers of amplified loci that are generated [40]. Additionally, PCoA analysis (Fig. 2) confirmed the distribution of the studied genotypes into three groups, a separation likely related to the reduced gene flow between the groups [41] as demonstrated by the values of *Nm* (1.3–1.6) obtained for *Lippia* species.

However, Bayesian analysis performed using the program STRUCTURE identified only two genetic groups (*K *=2), demonstrating that *L. lupulina* shares 50% of the genome of each group (Fig. 3), for more detail see Additional file 1. This result corroborates the results of Campos et al., [18], which classified Rhodolippia section (Group 2) as an intermediary between *Zapania* (Group 1) and *Goniostachyum* (Group 3) sections.

A recent study by O’Leary et al. [17] grouped *L. origanoides* × *velutina*, *L. velutina*, *L. sidoides*, and *L. salviifolia*, but not *L. grata*, within *L. origanoides*. Our results showed that individuals classified as *L. origanoides*, *L. origanoides* × *velutina*, *L. velutina*, *L. sidoides*, *L. salviifolia*, and *L. grata* formed a single group due to their strong genetic similarity, and therefore should be recognized as a single taxon to be named *L. origanoides*.

### Nuclear ribosome ITS2

The results presented herein show that species in the genus *Glandularia* and *Junellia* may be considered genetically similar as were forming one group (Fig. 4), thus confirming former results [42]. Furthermore, the species used as an outgroup, *Phyla* canescens, showed clear genetic divergence from *Lantana*, *Glandularia*, *Junellia* and *Lippia*, even though the separation of these genus has been proposed based on increased morphological descriptors [43, 44].

*Lippia alba* and *L. lupulina* are closely related to members of the genus *Lantana* and, together, they can be considered sister-groups [45–47], attesting the genetic similarity between the genera *Lippia* and *Lantana* [18, 48, 49].

Additionally, *L. alba* and *L. lupulina* exhibit longer branches in comparison with other *Lippia* species, suggesting that they underwent a more accelerated evolutionary rate and that they are older species [20, 43, 50].

The results of the phylogenetic analysis performed with ITS2 markers confirmed the results obtained with AFLP markers, suggesting the existence of only three species, namely *L. alba*, *L. lupulina* and *L. origanoides*. Of these, *L. alba* (section *Zapania*) can be considered the most divergent within the genus, whereas *L. lupulina* (section *Rhodolippia*) represents an intermediate between sections *Zapania* and *Goniostachyum,* for more detail see Additional files 2 and 3. In this aspect, the findings from the molecular-based analyses corroborate those based on cytogenetic and morphological characteristics [15, 16, 18].

### Chemical markers

The PCA analysis of the terpenoid composition from *L. origanoides L. origanoides* × *velutina*, *L. velutina*, *L. sidoides*, *L. salviifolia* and *L. grata* showed no specific grouping by species (Fig. 5), suggesting that they are different chemotypes. Conversely, Sandasi et al. [51], when investigating the chemotaxonomic differentiation of South-African *Lippia* species, namely *L. javanica*, *L. scaberrima*, *L. rehmannii* and *L.* wilmsii, were able to separate the species into distinct clusters. These results, paired with AFPL and ITS, suggest that *L. origanoides*, *L. origanoides* × *velutina*, *L. velutina*, *L. sidoides*, *L. salviifolia,* and *L. grata* belong to the same species, but present different chemotypes, for more detail see Additional file 4.

The chemotypes may be associated with the diverse biotic and abiotic stimuli to which each of the individuals had been subjected, which led to the creation of a complex biological system [52]. It is clear that nowadays the taxonomic identification of plants frequently rely on molecular biology techniques, especially when plants exhibit very similar morphological characters. In regards to medicinal plants, the use of chemical markers becomes essential if we consider that the biological activity can, most of the time, be related to a specific chemotype. Therefore, when any species is employed in the production of certified phytomedicines, the plant material must be identified taxonomically and the chemotype identified to assure the biological activity of the extract.

## Conclusions

The molecular markers AFLP and ITS2 were efficient in separating *L. alba* and *L. lupulina*, and in grouping *L. origanoides*, *L. origanoides* × *velutina*, *L. velutina*, *L. sidoides*, *L. salviifolia,* and *L. grata*. Moreover, the markers revealed the existence of intra- and inter-specific variability within the genus, as well as the close phylogenetic relationship between *L. alba* and *L. lupulina*. Since individuals grouped in *L. origanoides* exhibit morphological diversity and variability regarding the major constituents of the essential oils, the selection of genotypes for the production of certified phytomedicines must be based on the chemical profile of the oil produced.

## Additional files

**Additional file 1: Table S1.** Binary data.

**Additional file 2: Table S2.** Accession number of ITS2 nucleotide sequence from GenBank database at the National Center for Biotechnology Information (NCBI), for all species used as reference.

**Additional file 3: Table S3.** Fasta Sequences of amplified ITS fragments for all samples.

**Additional file 4: Table S4.** Chemical data.

## Abbreviations

LT: individuals from the medicinal plants germplasm bank (Ribeirão Preto University, Brazil)
LU: individuals from medicinal botanical garden of Nature Pharmacy, Brazil
