# Supplementary material for: Genetic diversity and chemical variability of Lippia spp. (Verbenaceae)
Source: BMC Res Notes. 2018 Oct 12;11:725. doi: 10.1186/s13104-018-3839-y (PMC6186075; doi:10.1186/s13104-018-3839-y)
Supplement: Supplementary file 2 — Additional file 2: Table S2. Accession number of ITS2 nucleotide sequence from GenBank database at the National Center for Biotechnology Information (NCBI), for all species used as reference. [file 13104_2018_3839_MOESM2_ESM.docx]

**Table S2**

Accession number of ITS2 nucleotide sequence from GenBank database at the National Center for Biotechnology Information (NCBI), for all species used as reference

| Species | Accession number |
| --- | --- |
| *Aloysia triphylla* | **EU761080.1** |
| *A. gratissima* | **DQ463782.1** |
| *A. gratissima var. schulziae* | **AY178651.1** |
| *Glandularia araucana* | **FJ867429.1** |
| *G. aristigera* | **FJ867424.1** |
| *G. aurantiaca* | **FJ867427.1** |
| *G. bipinnatifida* | **JN686504.1**, **FN867440.1** |
| *G. cheitmaniana* | **FJ867444.1** |
| *G. chiricahensis* | **FJ867436.1** |
| *G. dissecta* | **FJ867419.1** |
| *G. flava* | **FJ867428.1** |
| *G. gooddingii* | **FJ867439.1**, **FJ867437.1** |
| *G. guaranítica* | **FJ867434.1** |
| *G. mendocina* | **FJ867421.10** |
| *G. microphylla* | **FJ867432.1** |
| *G. scrobiculata* | **FJ867446.1** |
| *G. subincana* | **FJ867442.1** |
| *G. tenera* | **FJ867431.1** |
| *G. wrightii* | **AY928525.1** |
| *Junellia asparagoides* | **FJ867458.1** |
| *J. aspera* | **FJ867459.1** |
| *J. aspera var. longidentata* | **FJ867460.1** |
| *J. caespitosa* | **FJ867466.1** |
| *J. ligustrina var. lorentzii* | **FJ867468.1** |
| *J. micranta* | **FJ867462.1** |
| *J. selaginoides* | **FJ867463.1** |
| *J. spathulata* | **FJ867456.1** |
| *J. uniflora* | **FJ867450.1** |
| *Lantana angustifolia* | **HM120857.1** |
| *L. camara* | **AF437858.1** |
| *L. depressa var. floridana* | **FJ004802.1** |
| *L. hirsute* | **HM120856.1** |
| *L. hodgei* | **HM120851.1** |
| *L. horrid* | **HM120852.1**, **DQ463783.1** |
| *L. micrantha* | **HM120854.1** |
| *L. scaabrida* | **HM120860.1** |
| *L. strigocamara* | **FJ004800.1**, **HM120861.1** |
| *L. urticoides* | **AY178664.1** |
| *Lantana sp.* | **HM120855.1**, **EF190037.1** |
| *Lippia alba* | **EU761076.1**, **EU761078.1** |
| *Nashia inaguensis* | **DQ070737.1** |
| *Phyla canescens* | **HM193969.1**, **HM193970.1**, **193941.1**, **HM193957.1,** **HM193965.1**, **HM193967.1**, **HM194007.1**, **HM193962.1,** |
| *P. dulcis* | **EU761079.1** |
| *P. nodiflora* | **JN705432.1**, **HM194057.1**, **HM194070.1**, **HM194077.1**, **JQ014151.1**, **HM194044.1**, **HM194051.1**, **JN705431.1**, **JN705400.1**, **HM194153.1**, **HM194073.1**,**HM194082.1**, **HM194123.1**, **HM194133.1**, **HM194137.1**, **HM194115.1** **HM194117.1**, **HM194140.1** |
| *Phyla sp.* DAR14 | **HM194019.1** |
| *Premna serratifolia* | **DQ070745.1** |
| *Rhaphithamnus spinosus* | **DQ070743.1** |
| *Verbena bracteata* | **FJ867408.1** |
| *V. officinalis* | **FJ980370.1** |
| *Verbena x hybrida* | **AF477792.1** |
