# Supplementary material for: Genetic diversity and chemical variability of Lippia spp. (Verbenaceae)
Source: BMC Res Notes. 2018 Oct 12;11:725. doi: 10.1186/s13104-018-3839-y (PMC6186075; doi:10.1186/s13104-018-3839-y)
Supplement: Supplementary file 3 — Additional file 3: Table S3. Fasta Sequences of amplified ITS fragments for all samples. [file 13104_2018_3839_MOESM3_ESM.docx]

| **Code** | **Fragment (bp)** | **Sequence** |
| --- | --- | --- |
| LT1 | 202 | CGGACGACGCCGTCGCACGACAGTCGCATGAGTTGAGTGTTCAACCACCACTGGTCGTGACGTGCGTCGCCGGGGGATCGCATTTGGGCCAACCGCGCACGAGGGCGCACGGGAGGCCATTATCCGCCCCGCTCGCGCCCGTCCCCGTTCGGGGAGCGGGAGAGTGGGGCGACGCGATGCGTGACGCCCAGGCAGACGTGCC |
| LT2 | 204 | AGCGGACGACGCCGTCGCACGACAGTCGCATGAGTTGAGTGTACAACCACCACTGGTCGTGACGTGCGTCGCCGGGGGATCGCATTTGGGCCAACCGCGCACGCGAGCGCACGGGAGGCCATTATCCGCCCCGCTCGCGCCCGTCCCCGTTCGGGGAGCGGGAGAGTGGGGCGACGCGATGCGTGACGCCCAGGCAGACGTGCC |
| LT3 | 199 | GCGGACGACGCCGTCGCACGACAGTCGCATGAGTTGAGTGTTCAACCACCACTGGTCGTGACGTGCGTCGCCGGGGGATTCGCATTTGGGCCAACCGCGCACGAGGGCGCACGGGaGGCCATTATCCGCCCCGCTCGCGCCCGTCCCCGTTCGGGAGCGGGAGAGTGGGGCGACGCGATG  CGTGACGCCCAGGCAGACG |
| LT4 | 208 | CGAGCGGACGACGCCGTCGCACGACAGTCGCATGAGTTGAGTGTTCAACCACCACTGGTCGTGACGTGCGTCGCCGGGGGATCGCATTTGGGCCAACCGCGCACGCGAGCGCACGGGAGGCCATTATCCGCCCCGCTCGCGCCCGTCCCCGTTCGGGAGCGGGAGAGTGGGGCGACGCGATGCGTGACGCGCCAGGCAGACGTGCCCT |
| LT5 | 292 | AATCCCGCCTGACCTGGGGTCGCGGTCGGAGTGCACGCACCATCGGGTCGTATGGATTCCCGAGCGGACGACGCCGTCGCACGACAGTCGCATGAGTTGAGTGTTCAACCACCACTGGTCGTGACGTGCGTCGCCGGGGGATCGCATTTGGGCCAACCGCGCACGCGAGCGCACGGGAGGCCATTATCCGCCCCGCTCGCGCCCGTCCCCGTTCGGGAGCGGGAGAGTGGGGCGACGCGATGCGTGACGCCCAGGCAGACGTGCCCTCGGCCTAATGGCTTCGGGCGCAACT |
| LT6 | 175 | GAGACGCCGTCGCACGACAGTCGCATGAGTTGAGTGTTCAACCACCACTGGTCGTGACGTGCGTCGCCGGGGGATCGCATTTGGGCCAACCGCGCACGAGGGCGCACGGGAGGCCATTATCCGCCCCGCTCGCGCCCGTCCCCGTTCGGGAGCGGGAGAGTGGGGCGACGCGATG |
| LT7 | 203 | GAGCGGACGACGCCGTCGCACGACAGTCGCATGAGTTGAGTGTTCAACCACCACTGGTCGTGACGTGCGTCGCCGGGGGATCGCATTTGGGCCAACCGCGCACGCGAGCGCACGGGAGGCCATTATCCGCCCCGCTCGCGCCCGTCCCCGTTCGGGGAGCGGGAGAGTGGGGCGACGCGA  TGCGTGACGCCCAGGCAGACGTG |
| LT8 | 194 | TCGCACCACAGTCGCATGAGTTGAGTGTTCAACCACCACTGGTCGTGACGTGCGTCGCCGGGGGATCGCATTTGGGCCAACCGCGCACGCAAGGCGCACGGGAGGCCATTATCCGCCCCGCTCGCGCCCGTCCCCGTTCGGGAAGCGGGGAGAGTGGGGCGACGCGATGCGtGACGCCCAGGCAGACGTGCCTC |
| LT9 | 204 | CGGACGACGCCGTCGCACGACAGTCGCATGAGTTGAGTGTTCAACCACCACTGGTCGTGACGTGCGTCGCCGGGGGATCGCATTTGGGCCAACCGCGCACGCAGGGCGCACGGGAGGCCATTATCCGCCCCGCTCGCGCCCGTCCCCGTTCGGGGAGCGGGAGAGTGGGGCGACGCGATGCGTGACGCCCAGGCAGACGTGCCC |
| LT10 | 197 | GACGACGCCGTCGCACGACAGTCGCATGAGTTGAGTGTTCAACCACCACTGGTCGTGACGTGCGTCGCCGGGGGATCGCATTTGGGCCAACCGCGCACGCGAGCGCACGGGAGGCCATTATCCGCCCCGCTCGCGCCCGTCCCCGTTCGGGGAGCGGGAGAGTGGGGCGACGCGATGcGT  GACGCCCAGGCAGACGT |
| LT11 | 196 | GACGACGCCGTCGCACGACAGTCGCATGAGTTGAGTGTTCAACCACCACTGGTCGTGACGTGCGTCGCCGGGGGATCGCATTTGGGCCAACCGCGCACGAGGGCGCACGGGAGGCCATTATCCGCCCCGCTCGCGCCCGTCCCCGTTCGGGGAGCGGGAGAGTGGGGCGACGCGATGCGT  GACGCCCAGGCAGACG |
| LT12 | 195 | ACGACGCCGTCGCACGACAGTCGCATGAGTTGAGTGTTGCAACCACCACTGGTCGTGACGTGCGTCGCCGGGGGATCGCATTTGGGCCAACCGCGCACGAGGGCGCACGGGAGGCCATTATCCGCCCCGCTCGCGCCCGTCCCCGTTCGGGGAGCGGGAGAGTGGGGCGACGCGATGCGT  GACGCCCAGGCAGAC |
| LT14 | 202 | AGCGGACGACGCCGTCGCACGACAGTCGCATGAGTTGAGTGTWCAACCACCACTGGTCGTGACGTGCGTCGCCGGGGGATCGCATTTGGGCCAACCGCGCACGCGAGCGCACGGGAGGCCATTATCCGCCCCGCTCGCGCCCCGTCCCCGTTCGGGGAGCGGGAGAGTGGGGCGACGCGA  TGCGTGACGCCCAGGCAGACGT |
| LT15 | 197 | GGACGACGCCGTCGCACGACAGTCGCATGAGTTGAGTGTTCAACCACCACTGGTCGTGACGTGCGTCGCCGGGGGATCGCATTTGGGCCAACCGCGCACGAGGGCGCACGGGAGGCCATTATCCGCCCCGCTCGCGCCCGTCCCCGTTCGGGGAGCGGGAGAGTGGGGCGACGCGATGCG  TGACGCCCAGGCAGACG |
| LT16 | 201 | TCGTATGGATTCCCTAGCGGACGACGCCGTCGCACGACAGTCGCATGAGTTGAGTGTACAACCACCACTGGTCGTGACGTGCGTCGCCGGGGGATCGCATTTGGGCCAACCGCGCACGCAGCGCACGGGAGGCCATTATCCGCCCCGCTCGCGCCCCGTCCCCGTTCGGGGAGCGGGAGA  GTGGGGCGACGCGATGCGTGA |
| LT19 | 200 | CCTGACCTGGGGTCGCGGTCGGAGTGCACGCACCATCGGGTCGTATGGATTCCCGAGCGGAGACGCCGTCGCACGACAGTCGCATGAGTTGAGTGTTCAACCACCACTGGTCGTGACGTGCGTCGCCGGGGGACTCGGCGATTTGGGCCAACCGCGCACCGCGAGCGCACGGGAGGCCATTATCCCGCCCCGCTCGCGCC |
| LT20 | 227 | GTATGGATTCCCGAGCGGACGACGCCGTCGCACGACAGTCGCATGAGTTGAGTGTTCAACCACCACTGGTCGTGACGTGCGTCGCCGGGGGATCGCATTTGGGCCAACCGCGCACGCGAGCGCACGGGAGGCCATTATCCGCCCCGCTCGCGCCCGTCCCCGTTCGGGGAGCGGGAGAGT  GGGGCGACGCGATGCGTGACGCCCAGGCAGACGTGCCCTCGGCCTAA |
| LT23 | 200 | GCGGTCGGAGGTGCACGCACCATCGGGTCGTATGGCATTCCCGAGCGGACGACGCCGTCGCACGACAGTCGGCATGAGTTGAGTGTTCAACCACCACTGGTCGTGACGTGCGTCGCCGGGGGATCGCATTTGGGCCAACCGCGCACGCAGGGCGCACGGGAGGCCATTATCCGCCCCGCTCGCGCCCGTCCCCGTTCGGG |
| LT24 | 216 | GAGCGGACGACGCCGTCGCACGACAGTCGCATGAGTTGAGTGTTCAACCACCACTGGTCGTGACGTGCGTCGCCGGGGGATCGCATTTGGGCCAACCGCGCACGAGGGCGCACGGGAGGCCATTATCCGCCCCGCTCGCGCCCGTCCCCGTTCGGGGAGCGGGAGAGTGGGGCGACGCGA  TGCGTGACGCCCAGGCAGACGTGCCCTCGGCCTAAT |
| LT26 | 194 | ACGCCGTCGCACGACAGTCGCATGAGTTGAGTGTTCAACCACCACTGGTCGTGACGTGCGTCGCCGGGGGATCGCATTTGGGCCAACCGCGCACGCGAGCGCACGGGAGCCATTATCCGCCCCGCTCGCGCCCGTCCCCGTTCGGGAGCGGGAGAGTGGGGCGACGCGATGCGTGACGCC  CAGGCAGACGTGCC |
| LT27 | 214 | AGCGGACGACGCCGTCGCACGACAGTCGCATGAGTTGAGTGTTCAACCACCACTGGTCGTGACGTGCGTCGCCGGGGGATCGCATTTGGGCCAACCGCGCACGCGAGCGCACGGGAGGCCATTATCCGCCCCGCTCGCGCCCGTCCCCGTTCGGGGAGCGGGAGAGTGGGGCGACGCGATGCGTGACGCCCAGGCAGACGTGCCCTCGGCCTAA |
| LT30 | 203 | ACGACGCCGTCGCACGACAGTCGCATGAGTTGAGTGTTCAACCACCACTGGTCGTGACGTGCGTCGCCGGGGGATCGCATTTGGGCCAACCGCGCACGAGGGCGCACGGGAGGCCATTATCCGCCCCGCTCGCGCCCGTCCCCGTTCGGGGAGCGGGAGAGTGGGGCGACGCGATGCGTGACGCCCAGGCAGACGTGCCCTCG |
| LT31 | 197 | ACGCCGTCGCACGACAGTCGCATGAGTTGAGTGTACAACCACCACTGGTCGTGACGTGCGTCGCCGGGGGATCGCATTTGGGCCAACCGCGCACGCGAGCGCACGGGAGGCCATTATCCGCCCCGCTCGCGCCCGTCCCCGTTCGGGGAGCGGGAGAGTGGGGCGACGCGATGCGTGACG  CCCAGGCAGACGTGCCC |
| LT32 | 162 | CAACCACCACTGGTCGTGACGTGCGTCGCCGGGGGATCGCATTTGGGCCAACCGCGCACGCGAGCGCACGGGAGGCCATTATCCGCCCCGCTCGCGCCCGTCCCCGTTCGGGGAGCGGGAGAGTGGGGCGACGCGATGCGTGACGCCCAGGCAGACGTGCCC |
| LT33 | 205 | AGCGGAGACGCCGTCGCACGACAGTCGCATGAGTTGAGTGTACAACCACCACTGGTCGTGACGTGCGTCGCCGGGGGATCGCATTTGGGCCAACCGCGCACGCGAGCGCACGGGAGGCCATTATCCGCCCCGCTCGCGCCCGTCCCCGTTCGGGGAAGCGGGAGAGTGGGGCGACGCGATGCGTGACGCCCAGGCAGACGTGCCC |
| LT34 | 205 | AGCGGACGACGCCGTCGCACGACAGTCGCATGAGTTGAGTGTACAACCACCACTGGTCGTGACGTGCGTCGCCGGGGGATCGCATTTGGGCCAACCGCGCACGCGAGCGCACGGGAGGCCATTATCCGCCCCGCTCGCGCCCGTCCCCGTTCGGGGAGCGGGAGAGTGGGGCGACGCGATGCGTGACGCCCAGGCAGACGTGCCC |
| LT35 | 202 | AGCGGACGACGCCGTCGCACGACAGTCGCATGAGTTGAGTGTTCAACCACCACTGGTCGTGACGTGCGTCGCCGGGGGATCGCATTTGGGCCAACCGCGCACGAGGGCGCACGGGAGGCCATTATCCGCCCCGCTCGCGCCCGTCCCCGTTCGGGGAGCGGGAGAGTGGGGCGACGCGATGCGTGACGCCCAGGCAGACGTG |
| LT36 | 155 | AGCGGACGACGCCGTCGCACGACAGTCGCATGAGTTGAGTGTACAACCACCACTGGTCGTGACGTGCGTCGCCGGGGGATCGCATTTGGGCCAACCGCGCACGCGAGCGCACGGGAGGCCATTATCCGCCCCGCTCGCGCCCGTCCCCGTTCGGG |
| LT38 | 154 | AGCGGACACGCCGTCGCACGACAGTCGCATGAGTTGAGTGTACAACCACCACTGGTCGTGACGTGCGTCGCCGGGGGATCGCATTTGGGCCAACCGCGCACGAAGCGCACGGGAGGCCATTATCCGCCCCGCTCGCGCCCCGTCCCCGTTCGGG |
| LT42 | 203 | CCGTCGCACGACAGTCGCATGAGTTGAGTGTTCAACCACCACTGGTCGTGACGTGCGTCGCCGGGGGATCGCATTTGGGCCAACCGCGCACGCGAGCGCACGGGAGGCCATTATCCGCCCCGCTCGCGCCCGTCCCCGTTCGGGGAGCGGGAGAGTGGGGCGACGCGATGCGTGACGCCCAGGCAGACGTGCCCTCGGCCTAA |
| LT43 | 201 | GCGGACGACGCCGTCGCACGACAGTCGCATGAGTTGAGTGTTCAACCACCACTGGTCGTGACGTGCGTCGCCGGGGGATCGCATTTGGGCCAACCGCGCACGAGGGCGCACGGGAGGCCATTATCCGCCCCGCTCGCGCCCGTCCCCGTTCGGGGAGCGGGAGAGTGGGGCGACGCGATGCGTGACGCCCAGGCAGACGTG |
| LT44 | 201 | GCGGAGACGCCGTCGCACGACAGTCGCATGAGTTGAGTGTACAACCACCACTGGTCGTGACGTGCGTCGCCGGGGGATCGCATTTGGGCCAACCGCGCACGCGAGCGCACGGGAGGCCATTATCCGCCCCGCTCGCGCCCGTCCCCGTTCGGGGAAGCGGGAGAGTGGGGCGACGCGATGCGTGACGCCCAGGCAGACGTG |
| LT45 | 193 | ACGCCGTCGCACGACAGTCGCATGAGTTGAGTGTTCAACCACCACTGGTCGTGACGTGCGTCGCCGGGGGATCGCATTTGGGCCAACCGCGCACGAGGGCGCACGGGAGGCCATTATCCGCCCCGCTCGCGCCCGTCCCCGTTCGGGAGCGGGAGAGTGGGGCGACGCGATGCGTGACGCCCAGGCAGACGTG |
| LT46 | 160 | AGCGGACGACGCCGTCGCACGACAGTCGCATGAGTTGAGTGTACAACCACCACTGGTCGTGACGTGCGTCGCCGGGGGATCGCATTTGGGCCAACCGCGCACGCGAGCGCACGGGAGGCCATTATCCGCCCCGCTCGCGCCCGTCCCCGTTCGGGCGTGC |
| LT47 | 202 | AGCGGACGACGCCGTCGCACGACAGTCGCATGAGTTGAGTGTACAACCACCACTGGTCGTGACGTGCGTCGCCGGGGGATCGCATTTGGGCCAACCGCGCACGCGAGCGCACGGGAGGCCATTATCCGCCCCGCTCGCGCCCGTCCCCGTTCGGGGAGCGGGAGAGTGGGGCGACGCGATGCGTGACGCCCAGGCAGACGTG |
| LT48 | 201 | GCGGACGACGCCGTCGCACGACAGTCGCATGAGTTGAGTGTTCAACCACCACTGGTCGTGACGTGCGTCGCCGGGGGATCGCATTTGGGCCAACCGCGCACGAGGGCGCACGGGAGGCCATTATCCGCCCCGCTCGCGCCCGTCCCCGTTCGGGGAGCGGGAGAGTGGGGCGACGCGATGCGTGACGCCCAGGCAGACGTG |
| LT49 | 202 | CGGACGACGCCGTCGCACGACAGTCGCATGAGTTGAGTGTTCAACCACCACTGGTCGTGACGTGCGTCGCCGGGGGATCGCATTTGGGCCAACCGCGCACGAGGGCGCACGGGAGGCCATTATCCGCCCCGCTCGCGCCCGTCCCCGTTCGGGGAGCGGGAGAGTGGGGCGACGCGATGCGTGACGCCCAGGCAGACGTGCC |
| LT52 | 199 | GCGGAGACGCCGTCGCACGACAGTCGCATGAGTTGAGTGTTCAACCACCACTGGTCGTGACGTGCGTCGCCGGGGGATCGCATTTGGGCCAACCGCGCACGCGAGCGCACGGGAGGCCATTATCCGCCCCGCTCGCGCCCGTCCCCGTTCGGGAGCGGGAGAGTGGGGCGACGCGATGCGTGACGCCCAGGCAGACGTG |
| LT53 | 194 | ACGCCGTCGCACGACAGTCGCATGAGTTGAGTGTTCAACCACCACTGGTCGTGACGTGCGTCGCCGGGGGATCGCATTTGGGCCAACCGCGCACGCGAGCGCACGGGAGGCCATTATCCGCCCCGCTCGCGCCCGTCCCCGTTCGGGGAGCGGGAGAGTGGGGCGACGCGATGCGTGACGCCCAGGCAGACGTG |
| LT54 | 198 | CGGAGACGCCGTCGCACGACAGTCGCATGAGTTGAGTGTTCAACCACCACTGGTCGTGACGTGCGTCGCCGGGGGATCGCATTTGGGCCAACCGCGCACGAGGGCGCACGGGAGGCCATTATCCGCCCCGCTCGCGCCCGTCCCCGTTCGGGGAGCGGGAGAGTGGGGCGACGCGATGCGTGACGCCCAGGCAGACGT |
| LT55 | 203 | CGGGTCGTATGGATTCCCGAGCGGACGACGCCGTCGCACGACAGTCGCATGAGTTGAGTGTTCAACCACCACTGGCCGTGACGTGCGTCGCCGGGGGATCGCATTCGGGCCAACCGCGCACGCGAGCGCACGGGAGGCCATTATCCGCCCCGCTCGCGCCCGTCCCCATTCGGGGAGCGGGAGAGTGGGGCGACGCGATGCGT |
| LT57 | 202 | TCGGGTCGTATGGATTCCCGAGCGGACGACGCCGTCGCACGACAGTCGCATGAGTTGAGTGTTCAACCACCACTGGTCGTGACGTGCGTCGCCGGGGGATCGCATTTGGGCCAACCGCGCACGCGAGCGCACGGGAGGCCATTATCCGCCCCGCTCGCGCCCGTCCCCGTTCGGGGAGCGGGAGAGTGGGGCGACGCGATGC |
| LT59 | 201 | GGTCGCGGTCGGAGTGCACGCACCATCGGGTCGTATGGATTCCCGAGCGGACGACGCCGTCGCACGACAGTCGCATGAGTTGAGTGTTCAACCACCACTGGTCGTGACGTGCGTCGCCGGGGGATCGCATTTGGGCCAACCGCGCACGCAGGGCGCACGGGAGGCCATTATCCGCCCCGCTCGCGCCCCGTCCCCGTTCGG |
| LT60 | 202 | ATTCCCGAGCGGACGACGCCGTCGCACGACAGTCGCATGAGTTGAGTGTTCAACCACCACTGGTCGTGACGTGCGTCGCCGGGGGATCGCATTTGGGCCAACCGCGCACGCGAGCGCACGGGAGGCCATTATCCGCCCCGCTCGCGCCCGTCCCCGTTCGGGGAGCGGGAGAGTGGGGCGACGCGATGCGTGACGCCCAGGC |
| LT61 | 154 | GCGGACGACGCCGTCGCACGACAGTCGCATGAGTTGAGTGTTCAACCACCACTGGTCGTGACGTGCGTCGCCGGGGGATCGCATTTGGGCCAACCGCGCACGCGAGCGCACGGGAGGCCATTATCCGCCCCGCTCGCGCCCGTCCCCGTTCGGG |
| LT63 | 202 | GAGCGGACGACGCCGTCGCACGACAGTCGCATGAGTTGAGTGTTCAACCACCACTGGTCGTGACGTGCGTCGCCGGGGGATCGCATTTGGGCCAACCGCGCACGCGAGCGCACGGGAGGCCATTATCCGCCCCGCTCGCGCCCGTCCCCGTTCGGGGAGCGGGAGAGTGGGGCGACGCGA  TGCGTGACGCCCAGGCAGACGT |
| LT64 | 204 | CGGACGACGCCGTCGCACGACAGTCGCATGAGTTGAGTGTTCAACCACCACTGGTCGTGACGTGCGTCGCCGGGGGATCGCATTTGGGCCAACCGCGCACGAGGGCGCACGGGAGGCCATTATCCGCCCCGCTCGCGCCCGTCCCCGTTCGGGGAGCGGGAGAGTGGGGCGACGCGATGCGTGACGCCCAGGCAGACGTGCCCT |
| LT65 | 208 | GGACGACGCCGTCGCACGACAGTCGCATGAGTTGAGTGTTCAACCACCACTGGTCGTGACGTGCGTCGCCGGGGGATCGCATTTGGGCCAACCGCGCACGCGAGCGCACGGGAGGCCATTATCCGCCCCGCTCGCGCCCGTCCCCGTTCGGGGAGCGGGAGAGTGGGGCGACGCGATGCGTGACGCCCAGGCAGACGTGCCCTCGGCC |
| LT66 | 206 | CGGACGACGCCGTCGCACGACAGTCGCATGAGTTGAGTGTTCAACCACCACTGGTCGTGACGTGCGTCGCCGGGGGATCGCATTTGGGCCAACCGCGCACGCGAGCGCACGGGAGGCCATTATCCGCCCCGCTCGCGCCCGTCCCCGTTCGGGGAGCGGGAGAGTGGGGCGACGCGATGCGTGACGCCCAGGCAGACGTGCCCTCG |
| LT67 | 203 | GACGACGCCGTCGCACGACAGTCGCATGAGTTGAGTGTTCAACCACCACTGGTCGTGACGTGCGTCGCCGGGGGATCGCATTTGGGCCAACCGCGCACGAGGGCGCACGGGAGGCCATTATCCGCCCCGCTCGCGCCCGTCCCCGTTCGGGGAGCGGGAGAGTGGGGCGACGCGATGCGTGACGCCCAGGCAGACGTGCCCTC |
| LT68 | 202 | GTCGCACGACAGTCGCATGAGTTGAGTGTTCAACCACCACTGGTCGTGACGTGCGTCGCCGGGGGATCGCATTTGGGCCAACCGCGCACGCGAGCGCACGGGAGGCCATTATCCGCCCCGCTCGCGCCCGTCCCCGTTCGGGGAGCGGGAGAGTGGGGCGACGCGATGCGTGACGCCCAGGCAGACGTGCCCTCGGCCTAAT |
| LT69 | 204 | ATCGGGTCGTATGGATTCCCGAGCGGACGACGCCGTCGCACGACAGTCGCATGAGTTGAGTGTTCAACCACCACTGGTCGTGACGTGCGTCGCCGGGGGATCGCATTTGGGCCAACCGCGCACGCGAGGCGCACGGGAGGCCATTATCCGCCCCGCTCGCGCCCGTCCCCGTTCGGGGAGCGGGAGAGTGGGGCGACGCGATGC |
| LT70 | 217 | GGACGACGCCGTCGCACGACAGTCGCATGAGTTGAGTGTTCAACCACCACTGGTCGTGACGTGCGTCGCCGGGGGATCGCATTTGGGCCAACCGCGCACGCGAGCGCACGGGAGGCCATTATCCGCCCCGCTCGCGCCCGTCCCCGTTCGGGGAGCGGGAGAGTGGGGCGACGCGATGCGTGACGCCCAGGCAGACGTGCCCTCGGCCTAATGGCTT |
| LT71 | 206 | GGACGACGCCGTCGCACGACAGTCGCATGAGTTGAGTGTTCAACCACCACTGGTCGTGACGTGCGTCGCCGGGGGATCGCATTTGGGCCAACCGCGCACGCGAGCGCACGGGAGGCCATTATCCGCCCCGCTCGCGCCCGTCCCCGTTCGGGGAGCGGGAGAGTGGGGCGACGCGATGCGTGACGCCCAGGCAGACGTGCCCTCGG |
| LT72 | 208 | CGGACGACGCCTCGCACGACAGTCGCATGAGTTGAGTGTTCAACCACCACTGGTCGTGACGTGCGTCGCCGGGGGATCGCATTTGGGCCAACCGCGCACGAGGGCGCACGGGAGGCCATTATCCGCCCCGCTCGCGCCCGTCCCCGTTCGGGGAGCGGGAGAGTGGGGCGACGCGATGCGTGACGCCCAGGCAGACGTGCCCTCGGCC |
| LT73 | 201 | GCGGACGACGCCGTCGCACGACAGTCGCATGAGTTGAGTGTTCAACCACCACTGGTCGTGACCGTGCGTCGCCGGGGGATCGCATTTGGGCCAACCGCGCACGCGAGCGCACGGGAGGCCATTATCCGCCCCGCTCGCGCCCGTCCCCGTTCGGGAGCGGGAGAGTGGGGCGACGCGATGCGTGACGCCCAGGCAGACGTG |
| LT75 | 219 | CGGACGACGCCGTCGCACGACAGTCGCATGAGTTGAGTGTTCAACCACCACTGGTCGTGACGTGCGTCGCCGGGGGATCGCATTTGGGCCAACCGCGCACGCGAGCGCACGGGAGGCCATTATCCGCCCCGCTCGCGCCCGTCCCCGTTCGGGGAGCGGGAGAGTGGGGCGACGCGATGCGTGACGCCCAGGCAGACGTGCCCTCGGCCTAATGGCTTC |
| LT76 | 223 | GTGCACGCACCATCGGGTCGTATGGATTCCCGAGCGGACGACGCCGTCGCACGACAGTCGCATGAGTTGAGTGTTCAACCACCACTGGTCGTGACGTGCGTCGCCGGGGGATCGCATTTTGGGCCAACCGCGCACGCAAGCGCACGGGAGGCCATTATCCGCCCCGCTCGCGCCCGTCCC  CGTTCGGGAGCGGGAGAGTGGGGCGACGCGATGCGTGACGCCC |
| LT77 | 203 | GCGGACGACGCCGTCGCACGACAGTCGCATGAGTTGAGTGTTCAACCACCACTGGTCGTGACGTGCGTCGCCGGGGGATCGCATTTGGGCCAACCGCGCACGCGAGCGCACGGGAGGCCATTATCCGCCCCGCTCGCGCCCGTCCCCGTTCGGGGAGCGGGAGAGTGGGGCGACGCGATGCGTGACGCCCAGGCAGACGTGCC |
| LT78 | 197 | ATCGGGTCGTATGGATTCCCGAGCGGACGACGCCGTCGCACGACAGTCGCATGAGTTGAGTGTTCAAGCCACCACTGGTCGTGACGTGCGTCGCCGGGGGATCGCATTTGGGCCAACCGCGCACGCGAGCGCACGGGAGGCCATTATCCGCCCCGCTCGCGCCCGTCCCCGTTCGGGGAGCGGGAGAGTGGGGCGAC |
| LT79 | 201 | GCGGACGACGCCGTCGCACGACAGTCGCATGAGTTGAGTGTTCAACCACCACTGGTCGTGACGTGCGTCGCCGGGGGATCGCATTTGGGCCAACCGCGCACGCGAGCGCACGGGAGGCCATTATCCGCCCCGCTCGCGCCCGTCCCCGTTCGGGGAGCGGGAGAGTGGGGCGACGCGATGCGTGACGCCCAGGCAGACGTG |
| LT80 | 219 | AGCGGACGACGCCGTCGCACGACAGTCGCATGAGTTGAGTGTTCAACCACCACTGGTCGTGACGTGCGTCGCCGGGGGATCGCATTTGGGCCAACCGCGCACGCGAGCGCACGGGAGGCCATTATCCGCCCCGCTCGCGCCCGTCCCCGTTCGGGGAGCGGGAGAGTGGGGCGACGCGATGCGTGACGCCCAGGCAGACGTGCCCTCGGCCTAATGGCT |
| LT81 | 169 | CGTGCACGACAGTCGCATGAGTTGAGTGTTCAACCACCACTGGTCGTGACGTGCGTCGCCCGGGGGATCGCATTTGGGCCAACCGCGCACGCGAGGCGCACGGGAGGCCATTATCCGCCCCGCTCGCGCCCGTCCCCGTTCGGGAAGCGGGAGAGTGGGGCGACGCGAT |
| LT82 | 202 | AGCGGACGACGCCGTCGCACGACAGTCGCATGAGTTGAGTGTTCAACCACCACTGGTCGTGACGTGCGTCGCCGGGGGATCGCATTTGGGCCAACCGCGCACGCGAGCGCACGGGAGGCCATTATCCGCCCCGCTCGCGCCCGTCCCCGTTCGGGGAGCGGGAGAGTGGGGCGACGCGATGCGTGACGCCCAGGCAGACGTG |
| LT83 | 200 | AGCGGACGACGCCTCGCACGACAGTCGCATGAGTTGAGTGTTCAACCACCACTGGTCGTGACGTGCGTCGCCGGGGGATCGCATTTGGGCCAACCGCGCACGCAAGCGCACGGGAGGCATTATCCGCCCCGCTCGCGCCCGTCCCCGTTCGGGGAGCGGGAGAGTGGGGCGACGCGATGCGTGACGCCCAGGCAGACGTG |
| LT86 | 204 | TCGTATGGATTCCCGAGCGGACGACGCCGTCGCACGACAGTCGCATGAGTTGAGTGTTCAACCACCACTGGTCGTGACGTGCGTCGCCGGGGGATCGCATTTGGGCCAACCGCGCACGAGGGCGCACGGGAGGCCATTATCCGCCCCGCTCGCGCCCGTCCCCGTTCGGGGAGCGGGAGAGTGGGGCGACGCGATGCGTGACGC |
| LT87 | 202 | GAGCGGACGACGCCGTCGCACGACAGTCGCATGAGTTGAGTGTTCAACCACCACTGGTCGTGACGTGCGTCGCCGGGGGATCGCATTTGGGCCAACCGCGCACGCGAGCGCACGGGAGGCCATTATCCGCCCCGCTCGCGCCCGTCCCCGTTCGGGGAGCGGGAGAGTGGGGCGACGCGA  TGCGTGACGCCCAGGCAGACGT |
| LT88 | 204 | AGCGGACGACGCCGTCGCACGACAGTCGCATGAGTTGAGTGTTCAACCACCACTGGTCGTGACGTGCGTCGCCGGGGGATCGCATTTGGGCCAACCGCGCACGCAGGGCGCACGGGAGGCCATTATCCGCCCCGCTCGCGCCCGTCCCCGTTCGGGGAGGCGGGAGAGTGGGGCGACGCGATGCGTGACGCCCAGGCAGACGTG |
| LT89 | 204 | CGGACGACGCCGTCGCACGACAGTCGCATGAGTTGAGTGTTCAACCACCACTGGTCGTGACGTGCGTCGCCGGGGGATCCGCATTTGGGCCAACCGCGCACGAGGCGCACGGGAGGCCATTATCCGCCCCGCTCGCGCCCGTCCCCGTTCGGGGAGCGGGAGAGTGGGGCGACGCGATGCGTGACGCCCAGGCAGACGTGCCCT |
| LT90 | 213 | ATTCCCGAGCGGACGACGCCGTCGCACGACAGTCGCATGAGTTGAGTGTTCAACCACCACTGGTCGTGACGTGCGTCGCCGGGGGATCGCATTTGGGCCAACCGCGCACGCAGGGCGCACGGGAGGCCATTATCCGCCCCGCTCGCGCCCGTCCCCGTTCGGGGAGCGGGAGAGTGGGGCGACGCGATGCGTGACGCCCAGGCAGACGTGCCC |
| LT92 | 201 | CGGACGACGCCGTCGCACGACAGTCGCATGAGTTGAGTGTTCAACCACCACTGGTCGTGACGTGCGTCGCCGGGGGATCGCATTTGGGCCAACCGCGCACGCAGGGCGCACGGGAGGCCATTATCCGCCCCGCTCGCGCCCGTCCCCGTTCGGGGAGCGGGAGAGTGGGGCGACGCGATGCGTGACGCCCAGGCAGACGTG |
| LT93 | 201 | GCACGCACCATCGGGTCGTATGGATTCCCGAGCGGAGACGCCGTCGCACGACAGTCGCATGAGTTGAGTGTTCAACCACCACTGGTCGTGACGTGCGTCGCCGGGGGATCGCATTTGGGCCAACCGCGCACGCAGCGCACGGGAGGCCATTATCCGCCCCGCTCGCGCCCGTCCCCGTTCGGGGAGCGGGAGAGTGGGGCG |
| LT94 | 202 | GCGGACGACGCCGTCGCACGACAGTCGCATGAGTTGAGTGTTCAACCACCACTGGTCGTGACGTGCGTCGCCGGGGGATCGCATTTGGGCCAACCGCGCACGCGAGCGCACGGGAGGCCATTATCCGCCCCGCTCGCGCCCGTCCCCGTTCGGGGAGCGGGAGAGTGGGGCGACGCGATGCGTGACGCCCAGGCAGACGTGC |
| LT97 | 169 | TTCAACCACCACTGGTCGTGACGTGCGTCGCCGGGGGATCGCATTTGGGCCAACCGCGCACGCGAGCGCACGGGAGGCCATTATCCGCCCCGCTCGCGCCCGTCCCCGTTCGGGGAGCGGGAGAGTGGGGCGAGCGATGCGTGACGCCCAGGCAGACGTGCCCTCGGCC |
| LT98 | 203 | CGACGCCGTCGCACGACAGTCGCATGAGTTGAGTGTTCAACCACCACTGGTCGTGACGTGCGTCGCCGGGGGATCGCATTTGGGCCAACCGCGCACGCGAGCGCACGGGAGGCCATTATCCGCCCCGCTCGCGCCCGTCCCCGTTCGGGGAGCGGGAGAGTGGGGCGACGCGATGCGTGACGCCCAGGCAGACGTGCCCTCGG |
| LT99 | 191 | GCGTCGCCGGGGGATCGCATTTGGGCCAACCGCGCACGAGGGCGCACGGGAGGCCATTATCCGCCCCGCTCGCGCCCGTCCCCGTTCGGGGAGCGGGAGAGTGGGGCGACGCGATGCGTGACGCCCAGGCAGACGTGCCCTCGGCCTAATGGCTTCGGGCGCAACTTGCGTTCAAAGACTCGATGGTTCAC |
| LT100 | 199 | CATGAGTTGAGTGTTCAACCACCACTGGTCGTGACGTGCGTCGCCGGGGGATCGCATTTGGGCCAACCGCGCACGCGAGCGCACGGGAGGCCATTATCCGCCCCGCTCGCGCCCGTCCCCGTTCGGGGAGCGGGAGAGTGGGGCGACGCGATGCGTGACGCCCAGGCAGACGTGCCCTCGGCCTAATGGCTTCGGGCGC |
| LT101 | 199 | TCGGCATTTGGGGCCCAACCGCGCACGAGGGCGCACGGGAGGCCATTATCCGCCCCGCTCGCGGCCCCGTCCCCGTTCGGGGAGCGGGAGAGTGGGGCGGACCGCGATGCGTGACGCCCAGGCAGACGTGCCCTCGGCCTAATGGCTTCGGGCGCAACTTGCGTTCAAAGACTCGATGGTTCACGGGA |
| LT104 | 210 | GCGGACGACGCCGTCGCACGACAGTCGCATGAGTTGAGTGTTCAACCACCACTGGTCGTGACGTGCGTCGCCGGGGGATCGCATTTGGGCCAACCGCGCACGAGGGCGCACGGGAGGCCATTATCCGCCCCGCTCGCGCCCGTCCCCGTTCGGGGAGCGGGAGAGTGGGGCGACGCGATGCGTGACGCCCAGGCAGACGTGCCCTCGGCC |
| LT105 | 206 | ACGACGCCGTCGCACGACAGTCGCATGAGTTGAGTGTTCAACCACCACTGGTCGTGACGTGCGTCGCCGGGGGATCGCATTTGGGCCAACCGCGCACGCGAGCGCACGGGAGGCCATTATCCGCCCCGCTCGCGCCCGTCCCCGTTCGGGGAGCGGGAGAGTGGGGCGACGCGATGCGTGACGCCCAGGCAGACGTGCCCTCGGCC |
| LT107 | 204 | ACGCCGTCGCACGACAGTCGCATGAGTTGAGTGTTCAACCACCACTGGTCGTGACGTGCGTCGCCGGGGGATCGCATTTGGGCCAACCGCGCACGCGAGCGCACGGGAGGCCATTATCCGCCCCGCTCGCGCCCGTCCCCGTTCGGGGAGCGGGAGAGTGGGGCGACGCGATGCGTGACGCCCAGGCAGACGTGCCCTCGGCCT |
| LT108 | 206 | ACGACGCCGTCGCACGACAGTCGCATGAGTTGAGTGTTCAACCACCACTGGTCGTGACGTGCGTCGCCGGGGGATCGCATTTGGGCCAACCGCGCACGCGAGCGCACGGGAGGCCATTATCCGCCCCGCTCGCGCCCGTCCCCGTTCGGGGAGCGGGAGAGTGGGGCGACGCGATGCGTGACGCCCAGGCAGACGTGCCCTCGGCC |
| LT109 | 199 | ACAGTCGCATGAGTTGAGTGTTCAACCACCACTGGTCGTGACGTGCGTCGCCGGGGGATCGCATTTGGGCCAACCGCGCACGCGAGCGCACGGGAGGCCATTATCCGCCCCGCTCGCGCCCGTCCCCGTTCGGGGAGCGGGAGAGTGGGGCGACGCGATGCGTGACGCCCAGGCAGACGTGCCCTCGGCCTAATGGCTT |
| LT110 | 201 | ATGAGTTGAGAGTTCAACCACCACTGGTCGTGACGTGCGTCGCCGGGGGATCGCATTTGGGCCAACCGCGCACGCGAGCGCACGGGAGGCCATTATCCGCCCCGCTCGCGCCCCGTCCCCGTTGGGGGAAGCGGGAGAGTGGGGCGGACGCAATGCGTGACGCCCAGGCAGACGTGCCCTCGGCCTAATGGCTTCGGGCGC |
| LT111 | 202 | CGCACGACAGTCGCATGAGTTGAGTGTTCAACCACCACTGGTCGTGACGTGCGTCGCCGGGGGATCGCATTTGGGCCAACCGCGCACGAGGGCGCACGGGAGGCCATTATCCGCCCCGCTCGCGCCCGTCCCCGTTCGGGGAGCGGGAGAGTGGGGCGACGCGATGCGTGACGCCCAGGCAGACGTGCCCTCGGCCTAATGG |
| LT112 | 202 | CACGACAGTCGCATGAGTTGAGTGTTCAACCACCACTGGTCGTGACGTGCGTCGCCGGGGGATCGCATTTGGGCCAACCGCGCACGAGGGCGCACGGGAGGCCATTATCCGCCCCGCTCGCGCCCGTCCCCGTTCGGGGAGCGGGAGAGTGGGGCGACGCGATGCGTGACGCCCAGGCAGACGTGCCCTCGGCCTAATGGCT |
| LT113 | 200 | ATGAGTTGAGTGTTCAACCACCACTGGTCGTGACGTGCGTCGCCGGGGGATCGCATTTGGGCCAACCGCGCACGAGGGCGCACGGGAGGCCATTATCCGCCCCGCTCGCGCCCGTCCCCGTTCGGGGAGCGGGAGAGTGGGGCGACGCGATGCGTGACGCCCAGGCAGACGTGCCCTCGGCCTAATGGCTTCGGGCGCAA |
| LT114 | 199 | TTCCCGAGCGGACGACGCCGTCGCACGACAGTCGCATGAGTTGAGTGTTCAACCACCACTGGTCGTGACGTGCGGTCGCCGGGGGATCGCATTTGGGCCAACCGCGCACGAGGGCGCACGGGAGGCCATTATCCGCCCCGCTCGCGCCCGTCCCCGTTCGGGGAGCGGGAGAGTGGGGCGACGCGATGCGTGACGCCCA |
| LT115 | 197 | GTCGCACGACAGTCGCATGAGTTGAGTGTTCAACCACCACTGGTCGTGACGTGCGGTCGCCGGGGGATCGCATTTGGGCCAACCGCGCACGAGGGCGCACGGGAGGCCATTATCCGCCCCGCTCGCGCCCGTCCCCGTTCGGGGAGCGGGAGAGTGGGGCGACGCGATGCGTGACGCCCAGGCAGACGTGCCCTCGG |
| LT116 | 201 | GCGGACGACGCCGTCGCACGACAGTCGCATGAGTTGAGTGTTCAACCACCACTGGTCGTGACGTGCGTCGCCGGGGGATCGCATTTGGGCCAACCGCGCACGCGAGCGCACGGGAGGCCAtTTATCCGCCCCGCTCGCGCCCGTCCCCGTTCGGGAGCGGGAGAGTGGGGCGACGCGATGCGTGACGCCCAGGCAGACGTG |
| LT117 | 202 | TTCCCGAGCGGACGACGCCGTCGCACGACAGTCGCATGAGTTGAGTGTTCAACCACCACTGGTCGTGACGTGCGTCGCCGGGGGATCGCATTTGGGCCAACCGCGCACGCGAGCGCACGGGAGGCCATTATCCGCCCCGCTCGCGCCCGTCCCCGTTCGGGGAGCGGGAGAGTGGGGCGACGCGATGCGTGACGCCCAGGCA |
| LT118 | 202 | TGAGTTGAGAGTTCAAACCACCACTGGTCGTGACGTGCGTCGCCGGGGGATCGGCATTTGGGCCAACCGCGCACGCGAGCGCACGGGAGGCCATTATCCGCCCCGCTCGCGCCCGTCCCCGTTGGGGGAGCGGGAGAGTGGGGCGACGCAATGCGTGACGCCCAGGCAGACGTGCCCTCGGCCTAATGGCTTCGGGCGCAAC |
| LT120 | 202 | TTCCCGAGCGGACGACGCCGTCGCACGACGGTTGCGCGAGTTGAGTGTTCAACCACCAAAGGTCGTGACGTGCGTCGCCGAGGGATCGCATTTGGGCCGGCCGCGCGCTCTCGGCGCACGGGAGGCCATTATCCGCCCCGCTCGCGCCCGTTCCCTCGTGTATGGGGAGCGGGAGAGTGGGGCGACGCGATGCGTGACGCCC |
| LT121 | 202 | ACGCCGTCGCACGACGGTTGCGCGAGTTGAGTGTTCAACCACCAAAGGTCGTGACGTGCGTCGCCGAGGGATCGCATTTGGGCCGGCCGCGCGCTCTCGGCGCACGGGAGGCCATTATCCGCCCCGCTCGCGCCCGTTCCCTCATGTATGGGGAGCGGGAGAGTGGGGCGACGCGATGCGTGACGCCCAGGCAGACGTGCCC |
| LT122 | 203 | ATTCCCGAGCGGACGACGCGTCGCACGACGGTTGCGCGAGTTGAGTGTTCAACCACCAAAGGTCGTGACGTGCGTCGCCGAGGGATCGCATTTGGGCCGGCCGCGCGCTCTCGGCGCACGGGAGGCCATTATCCGCCCCGCTCGCGCCCGTTCCCTTGTGTATGGGGAGCGGGAGAGTGGGGCGACGCGATGCGTGACGCCCA |
| LT123 | 204 | TTGCGCGAGTTGAGTGTTCAACCACCAAAGGTCGTGACGTGCGTCGCCGAGGGATCGCATTTGGGCCGGCCGCGCGCTCTCGGCGCACGGGAGGCCATTATCCGCCCCGCTCGCGCCCGTTCCCTCGTGTATGGGGAGCGGGAGAGTGGGGCGACGCGATGCGTGACGCCCAGGCAGACGTGCCCTCGGCCTAATGGCTTCGGG |
| LT124 | 199 | GTCGCACGACGGTTGCGCGAGTTGAGTGTTCAACCACCAAAGGTCGTGACGTGCGTCGCCGAGGGATCGCATTTGGGCCGGCCGCGCGCTCTCGGCGCACGGGAGGCCATTATCCGCCCCGCTCGCGCCCGTTCCCTCGTGTATGGGGAGCGGGAGAGTGGGGCGACGCGATGCGTGACGCCCAGGCAGACGTGCCCTC |
| LT125 | 203 | GCGCGAGTTGAGTGTTCAACCACCAAAGGTCGTGACGTGCGTCGCCGAGGGATCGCATTTGGGCCGGCCGCGCGCTCTCGGCGCACGGGAGGCCATTATCCCGCCCCGCTCGCGCCCGTTCCCTCGTGTATGGGGAGCGGGAGAGTGGGGCGACGCGATGCGTGACGCCCAGGCAGACGTGCCCTCGGCCTAATGGCTTCGGG |
| LT126 | 204 | CGCGAGTTGAGTGTTCAACCACCAAAGGTCGTGACGTGCGTCGCCGAGGGATCGCATTTGGGCCGGCCGCGCGCTCTCGGCGCACGGGAGGCCATTATCCGCCCCGCTCGCGCCCGTTCCCTCGTGTATGGGGAGCGGGAGAGTGGGGCGACGCGATGCGTGACGCCCAGGCAGACGTGCCCTCGGCCTAATGGCTTCGGGCGC |
| LT127 | 203 | TGCGCGAGTTGAGTGTTCAACCACCAAAGGTCGTGACGTGCGTCGCCGAGGGATCGCATTTGGGCCGGCCGCGCGCTCTCGGCGCACGGGAGGCCATTATCCGCCCCGCTCGCGCCCGTTCCCTCGTGTATGGGGAGCGGGAGAGTGGGGCGACGCGATGCGTGACGCCCAGGCAGACGTGCCCTCTGCCTAATGGCTTCGGG |
| LT128 | 202 | GCGCGAGTTGAGTGTTCAACCACCAAAGGTCGTGACGTGCGTCGCCGAGGGATCGCATTTGGGCCGGCCGCGCGCTCTCTGCGCACGGGAGGCCATTATCCGCCCCGCTCGCGCCCGTTCCCTCGTGTATGGGGAGCGGGAGAGTGGGGCGACGCGATGCGTGACGCCCAGGCAGACGTGCCCTCGGCCTAATGGCTTCGGG |
| LU129 | 203 | ATTCCCGAGCGGACGACGCCGTCGCACGACAGTCGCATGAGTTGAGTGTTCAACCACCACTGGCCGTGACGTGCGTCGCCGGGGGATCGCATTTGGGCCAACCGCGCACGCGAGCGCACGGGAGGCCATTATCCGCCCCGCTCGCGCCCGTCCCCATTCGGGGAGCGGGAGAGTGGGGCGACGCGATGCGTGACGCCCAGGCA |
| LU130 | 204 | GCGGACGACGCCGTCGCACGACAGTCGCATGAGTTGAGTGTTCAACCACCACTGGTCGTGACGTGCGTCGCCGGGGGATCGCATTTGGGCCAACCGCGCACGCGAGCGCACGGGAGGCCATTATCCGCCCCGCTCGCGCCCGTCCCCGTTCGGGGAGCGGGAGAGTGGGGCGACGCGATGCGTGACGCCCAGGCAGACGTGCCC |
| LU132 | 203 | TATGGATTCCCGAGCGGACGACGCCGTCGCACGACAGTCGCATGAGTTGAGTGTTCAACCACCACTGGTCGTGACGTGCGTCGCCGGGGGATCGCATTTGGGCCAACCGCGCACGCGAGGCGCACGGGAGGCCATTATCCGCCCCGCTCGCGCCCGTCCCCAGTTCGGGGAGCGGGAGAG  TGGGGCGACGCGATGCGTGACGC |
| LU133 | 203 | GAGCGGACGACGCCGTCGCACGACAGTCGCATGAGTTGAGTGTTCAACCACCACTGGTCGTGACGTGCGTCGCCGGGGGATCGCATTTGGGCCAACCGCGCACGAGGGCGCACGGGAGGCCATTATCCGCCCCGCTCGCGCCCGTCCCCGTTCGGGGAGCGGGAGAGTGGGGCGACGCGATGCGTGACGCCCAGGCAGACGTG |
| LU134 | 197 | GTCGCACGACAGTCGCATGAGTTGAGTGTTCAACCACCACTGGTCGTGACGTGCGTCGCCGGGGGATCGCATTTGGGCCAACCGCGCACGAGGGCGCACGGGAGGCCATTATCCGCCCCGCTCGCGCCCGTCCCCGTTCGGGGAGCGGGAGAGTGGGGCGACGCGATGCGTGACGCCCAGGCAGACGTGCCCTCGGC |
| LU135 | 200 | CGCCGTCGCACGACAGTCGCATGAGTTGAGTGTTCAACCACCACTGGTCGTGACGTGCGTCGCCGGGGGATCGCATTTGGGCCAACCGCGCACGAGGGCGCACGGGAGGCCATTATCCGCCCCGCTCGCGCCCGTCCCCGTTCGGGGAGCGGGAGAGTGGGGCGACGCGATGCGTGACGCCCAGGCAGACGTGCCCTCGG |
| LU137 | 201 | ACGACGCCGTCGCACGACAGTCGCATGAGTTGAGTGTTCAACCACCACTGGTCGTGACGTGCGTCGCCGGGGGATCGCATTTGGGCCAACCGCGCACGCGAGCGCACGGGAGGCCATTATCCGCCCCGCTCGCGCCCGTCCCCGTTCGGGGAGCGGGAGAGTGGGGCGACGCGATGCGTGACGCCCAGGCAGACGTGCCCT |
| LU140 | 193 | AGTGTTCAcACCACCACTGGTCGTGACGTGCGTCGCCGGGGGATCGCATTTGGGCCAACCGCGCACGAGGGCGCACGGGAGGCCATTATCCGCCCCGCTCGCGCCCGTCCCCGTTCGGGGAGCGGGAGAGTGGGGCGACGCGATGCGTGACGCCCAGGCAGACGTGCCCTCGGCCTAATGGCTTCGGGCGCAA |
| LU141 | 198 | ACGACGCCGTCGCACGACAGTCGCATGAGTTGAGTGTTCAACCACCACTGGTCGTGACGTGCGTCGCCGGGGGATCGCATTTGGGCCAACCGCGCACGAGGGCGCACGGGAGGCCATTATCCGCCCCGCTCGCGCCCGTCCCCGTTCGGGGAGCGGGAGAGTGGGGCGACGCGATGCGTGACGCCCAGGCAGACGTGC |
| LU142A | 190 | CATGAGTTGAGAGTTAACCACCACTGGTCGTGACGTGGGTCGCCGGGGGTTGGCATTTGGGCCAACCGGGAAGGGAGCGCACGGGAGGATTATCCGCCCCGTCGCGCCCGTCCCGTTGGGGGAGCGGGAGAGTGGGGCGACGCGATGCGTGACGCCCAGGCAGACGTGCCTCGGCCTCAATGGCTTCGGG |
| LU142B | 199 | ACATGAGTTGAGAGTTCAACCACCACTGGTCGTGACGTGCGTCGCCGGGGGATCGCATTTGGGCCAACCGCGCACGCGAGCGCACGGGAGGCCATTATCCGCCCCGCTCGCGCCCGTTCCCGTTGGGGGAGCGGGAGAGTGGGGCGACGCGATGCGTGACGCCCAGGCAGACGTGCCCTCGGCCTAATGGCTTCGGGCG |
| LU143A | 195 | CATGAGTTGAGAGTTCAACCACCACTGGTCGTGACGTGCGTCGCCGGGGGTTCGCATTTGGGCCAACCGCGCACGCGAGCGCACGGGAGGCCATTATCCGCCCCGCTCGCGCCCGTTCCCGTTGGGGGAGCGGGAGAGTGGGGCGACGCGATGCGTGACGCCCAGGCAGACGTGCCCTCGGCCTAATGGCTTCGG |
| LU144B | 204 | GTCGCACGACAGTCGCATGAGTTGAGAGTTCAACCACCACTGGTCGTGACGTACGTCGCCGGGGGTTCGCATTTGGGCCAACCGCGCACGCGAGCGCACGGGAGGCCATTATCCGCCCCGCTCGCGCCCGTCCCCGTTGGGGGAGCGGGAGAGTGGGGCGACGCGATGCGTGACGCCCAGGCAGACGTGCCCTCGGCCTAATGG |
| LU145 | 204 | CCGTCGCACGACAGTCGCATGAGTTGAGAGTTCAACCACCACTGGTCGTGACGTGCGTCGCCGGGGGATCGCATTTGGGCCAACCGCGCACGCGAGCGCACGGGAGGCCATTATCCGCCCCGCTCGCGCCCGTCCCCGTTGGGGGAGCGGGAGAGTGGGGCGACGCAATGCGTGACGCCCAGGCAGACGTGCCCTCGGCCTAAT |
| LU146 | 200 | GACGCCGTCGCACGACAGTCGCATGAGTTGAGAGTTCAACCACCACTGGTCGTGACGTGCGTCGCCGGGGGATCGCATTTGGGCCAACCGCGCACGCGAGCGCACGGGAGGCCATTATCCGCCCCGCTCGCGCCCGTCCCCGTTGGGGGAGCGGGAGAGTGGGGCGACGCAATGCGTGACGCCCAGGCAGACGTGCCCTC |
| LU148 | 202 | CATGAGTTGAGAGTTCAACCACCACTGGTCGTGACGTGCGTCGCCGGGGGATCGCATTTGGGCCAACCGCGCACGCGAGCGCACGGGAGGCCCATTATCCGCCCCGCTCGCGCCCCGTCCCCGTTGGGGGAGCGGGAGAGTGGGGCGACGCAATGCGTGACGCCCAGGCAGACGTGCCCTCGGCCTAATGGCTTCGGGCGCA |
| LU151 | 204 | GCATGAGTTGAGTGTTCAACCACCACTGGTCGTGACGTGCGTCGCCGGGGGATCGCATTTGGGCCAACCGCGCACGCGAGCGCACGGGAGGCCATTATCCGCCCCGCTCGCGCCCGTCCCCGTTCGGGGAGCGGGAGAGTGGGGCGACGCGATGCGTGACGCCCAGGCAGACGTGCCCTCGGCCTAATGGCTTCGGGCGCAACT |
| LU153 | 200 | CACGACAGTCGCATGAGTTGAGTGTTCAACCACCACTGGTCGTGACGTGCGTCGCCGGGGGATCGCATTTGGGCCAACCGCGCACGCGAGCGCACGGGAGGCCATTATCCGCCCCGCTCGCGCCCGTCCCCGTTCGGGGAGCGGGAGAGTGGGGCGACGCGATGCGTGACGCCCAGGCAGACGTGCCCTCGGCCTAATGG |
| LU154 | 195 | ACGCCGTCGCACGACAGTCGCATGAGTTGAGTGTTCAACCACCACTGGTCGTGACGTGCGTCGCCGGGGGATCGCATTTGGGCCAACCGCGCACGCAGAGCGCACGGGAGGCCATTATCCGCCCCGCTCGCGCCCGTCCCCGTTCGGGGAGCGGGAGAGTGGGGCGACGCGATGCGTGACGCCCAGGCAGACGTG |
| LU155 | 204 | ACGCCGTCGCACGACAGTCGCATGAGTTGAGTGTTCAACCACCACTGGTCGTGACGTGCGTCGCCGGGGGATCGCATTTGGGCCAACCGCGCACGCGAGCGCACGGGAGGCCATTATCCGCCCCGCTCGCGCCCCGTCCCCGTTCGGGGAGCGGGAGAGTGGGGCGACGCGATGCGTGACGCCCAGGCAGACGTGCCCTCGGCC |
| LU156 | 188 | TTGAGTGTACAACCACCACTGGTCGTGACGTGCGTCGCCGGGGGATCGCATTTGGGCCAACCACGCACGCGAGCGCACGGGAGCCATTATCCGCCCCGCTCGCGCCCGTCCCCGTTCGGGGAGCGGGAGAGTGGGGCGACGCGATGCGTGACGCCCAGGCAGACGTCCCTCGGCCTAATGGCTTCGGG |
| LU157 | 200 | CATGAGTTGAGTGTTCAACCACCACTGGTCGTGACGTGCGTCGCCGGGGGATCGCATTTGGGCCAACCGCGCACGCAGAGCGCACGGGAGGCCATTATCCGCCCCGCTCGCGCCCGTCCCCGTTCGGGGAGCGGGAGAGTGGGGCGACGCGATGCGTGACGCCCAGGCAGACGTGCCCTCGGCCTAATGGCTTCGGGCGC |
| LU158 | 200 | GCATGAGTTGAGTGTTCAACCACCACTGGTCGTGACGTGCGTCGCCGGGGGATCGCATTTGGGCCAACCGCGCACGCGAGCGCACGGGAGGCCATTATCCGCCCCGCTCGCGCCCGTCCCCGTTCGGGGAGCGGGAGAGTGGGGCGACGCGATGCGTGACGCCCAGGCAGACGTGCCCTCGGCCTAATGGCTTCGGGCGC |
| LU159 | 201 | TTCCCGAGCGGACGACGCCGTCGCACGACAGTCGCATGAGTTGAGTGTTCAACCACCACTGGCCGTGACGTGCGTCGCCGGGGGATCGCATTTGGGCCAACCGCGCACGCGAGCGCACGGGAGGCCATTATCCGCCCCGCTCGCGCCCGTCCCCaTTCGGGGAGCGGGAGAGTGGGGCGACGCGATGCGTGACGCCCAGGC |
| LU163 | 201 | CGCCGTCGCACGACAGTCGCATGAGTTGAGTGTTCAACCACCACTGGTCGTGACGTGCGTCGCCGGGGGATCGCATTTGGGCCAACCGCGCACGCGAGCGCACGGGAGGCCATTATCCGCCCCGCTCGCGCCCCGTCCCCGTTCGGGGAAGCGGGAGAGTGGGGCGACGCGATGCGTGACGCCCAGGCAGACGTGCCCTCG |
| LU164 | 203 | ACAGTCGCATGAGTTGAGAGTTCAACCACCACTGGTCGTGACGTGCGTCGCCGGGGGATCGCATTTGGGCCAACCGCGCACGCGAGCGCACGGGAGGCCATTATCCGCCCCGCTCGCGCCCGTCCCCGTTGGGGGAGCGGGAGAGTGGGGCGACGCAATGCGTGACGCCCAGGCAGACGTGCCCTCGGCCTAATGGCTTCGGG |
| LU165 | 201 | CGTCGCACGACAGTTGCGAGAGTTGAGTGTTCAACCACCAAAGGTCGTGACGTGCGTCGCCGAGGGATGCCATTTGGGCCGGCCGCGCGCACTCGACGCACGGGAGGCCATTATCCGCCCCGCTCTCGCCCGTCCCCGTATGGGGAGAAGTAGAGTGGGGCGACGCGATGCGTGACGCCCAGGCAGACGTGCCCTCGGCCT |
